# Reference Genes for Real-Time PCR Quantification of Messenger RNAs and MicroRNAs in Mouse Model of Obesity

**DOI:** 10.1371/journal.pone.0086033

**Published:** 2014-01-17

**Authors:** Petra Matoušková, Hana Bártíková, Iva Boušová, Veronika Hanušová, Barbora Szotáková, Lenka Skálová

**Affiliations:** 1 Department of Biochemical Sciences, Charles University in Prague, Faculty of Pharmacy, Hradec Králové, Czech Republic; 2 Department of Medical Biology and Genetics, Charles University in Prague, Faculty of Medicine, Hradec Králové, Czech Republic; University of Catanzaro Magna Graecia, Italy

## Abstract

Obesity and metabolic syndrome is increasing health problem worldwide. Among other ways, nutritional intervention using phytochemicals is important method for treatment and prevention of this disease. Recent studies have shown that certain phytochemicals could alter the expression of specific genes and microRNAs (miRNAs) that play a fundamental role in the pathogenesis of obesity. For study of the obesity and its treatment, monosodium glutamate (MSG)-injected mice with developed central obesity, insulin resistance and liver lipid accumulation are frequently used animal models. To understand the mechanism of phytochemicals action in obese animals, the study of selected genes expression together with miRNA quantification is extremely important. For this purpose, real-time quantitative PCR is a sensitive and reproducible method, but it depends on proper normalization entirely. The aim of present study was to identify the appropriate reference genes for mRNA and miRNA quantification in MSG mice treated with green tea catechins, potential anti-obesity phytochemicals. Two sets of reference genes were tested: first set contained seven commonly used genes for normalization of messenger RNA, the second set of candidate reference genes included ten small RNAs for normalization of miRNA. The expression stability of these reference genes were tested upon treatment of mice with catechins using geNorm, NormFinder and BestKeeper algorithms. Selected normalizers for mRNA quantification were tested and validated on expression of NAD(P)H:quinone oxidoreductase, biotransformation enzyme known to be modified by catechins. The effect of selected normalizers for miRNA quantification was tested on two obesity- and diabetes- related miRNAs, miR-221 and miR-29b, respectively. Finally, the combinations of B2M/18S/HPRT1 and miR-16/sno234 were validated as optimal reference genes for mRNA and miRNA quantification in liver and 18S/RPlP0/HPRT1 and sno234/miR-186 in small intestine of MSG mice. These reference genes will be used for mRNA and miRNA normalization in further study of green tea catechins action in obese mice.

## Introduction

Obesity, disease characterized as a condition resulting from the excess accumulation of body fat, has become one of the most important public health problems worldwide. This condition has a large impact on several metabolic and chronic ailments including heart disease, arthritis, hypertension, hyperlipidemia, and type 2 diabetes [1]. Among other ways, nutritional intervention is important method for treatment and prevention of the obesity and metabolic syndrome [2].

Many phytochemicals, nonessential nutrients isolated from plants, have protective or disease-preventive properties. Green tea has been reported to possess preventive effects against a number of chronic diseases including heart disease, neurodegenerative disease and cancer [3], [4]. Furthermore, potential anti-obesity effect of green tea catechins has been widely studied (reviewed in [5]) and green tea extracts have become popular ingredients of many dietary supplements for weight reduction [6]. As these supplements usually contain concentrated phytochemicals, consumed doses exceed those that could be obtained from food. Therefore, apart from anticipated improvement of human health, it is essential to keep in mind possible undesired effects of these phytochemicals. While various effects of green tea catechins on healthy animals and human volunteers have been intensively studied (reviewed in [6]), information about their activities in obese animals/patients is missing.

To study the obesity in rodents, the animals after neonatal treatment with monosodium l-glutamate (MSG) are frequently used. In this model, obesity is caused mainly by the lack of physical activity and it is not accompanied by polyphagia [7]. Moreover, this model is characterized by hyperinsulinemia, hyperleptinemia, hyperglycaemia, insulin-resistance, and non-alcoholic fatty liver disease which makes it useful model of the diabetes and metabolic syndrome [8]–[10]. To understand the mechanism of certain phytochemical action in organism, the study of all regulatory levels is necessary. In addition to enzyme activities and corresponding protein amounts, the quantification of relevant mRNA and microRNA (miRNA) is extremely important.

The significance of miRNA, a class of small RNAs mediating post-transcriptional gene regulation, has been shown in several cellular processes as well as in different metabolic and pathologic conditions. MiRNAs typically bind to the 3′UTR of specific target mRNAs and suppress their translation or accelerate the degradation of the mRNA [11]. The expression, regulation and function of miRNA are addressed in an increasing number of studies. Subtle change in miRNA expression can have a significant impact on the cell biology [12]–[14]. Therefore, a proper normalization strategy that enables detection of these small changes is of the utmost importance.

Quantitative real-time RT-PCR (qPCR) is widely used as the most reliable method for quantifying gene transcript levels because of its sensitivity, accuracy and specificity [15]. However, it requires an appropriate internal reference gene (RG) to normalize the target gene expression. In theory, such internal control must exhibit constant expression levels in all cell types and experimental conditions. In fact, several studies have reported that stability of commonly used RGs can significantly vary in given experimental treatments and tissue types [16], [17]. Furthermore, it has been shown that the conventional use of single RG for normalization may lead to relatively large errors. Currently, the use of multiple internal control genes is considered as an essential approach for an accurate normalization of data [18]. Statistical algorithms such as geNorm [18], NormFinder [19] and BestKeeper [20], were developed to facilitate the evaluation of potential RG expression stability under different experimental conditions. In case of miRNA profiling, only few candidate reference miRNAs have been reported so far [21]–[23]. Generally, other small non-coding RNAs are used for normalization including 5S, small nuclear RNAs (U6) or small nucleolar RNAs (204, 234, U24, U26, RNU48).

Therefore, present study was designed to find out and evaluate the proper RGs for mRNA and miRNA expression profiling in MSG obese mice treated with green tea catechins. Our qPCR results were obtained in compliance with MIQE guidelines (MIQE checklist-S1) [15]. The selected RGs will be used for further study of mechanism of green tea catechins action in obese animals.

## Materials and Methods

### Ethic Statement

The mice were cared for and used in accordance with the Guide for the Care and Use of Laboratory Animals (Protection of Animals from Cruelty Act No. 246/92, Czech Republic). Ethical Committee of Charles University in Prague, Faculty of Pharmacy in Hradec Králové approved all animal experimental procedures (Permit Number: 34354/2010–30).

### Animals and Treatments

Male NMRI mice obtained from Bio Test (Konárovice, Czech Republic) were housed in air-conditioned animal quarters with a 12 h light/dark cycle at 23°C. Food (standard chow diet ST-1, Velaz, Koleč, Czech Republic) and tap water were provided *ad libitum*.

For hypothalamic lesion-induced obesity, MSG (4 mg/g body weight, s.c.) was administered to newborn mice daily from postnatal day 2 to 8 (from day 2 to 6 mice received 10 mg/day, 2 following days 20 mg/day). Controls were treated with saline of osmolality corresponding to the MSG solution.

At 7 months of age, mice were divided into 4 groups (11–13 mice per group): first group consisted of control (lean) mice with green tea catechins-enriched diet, second group consisted of MSG-treated (obese) mice with green tea catechins-enriched diet, lean and obese mice with standard diet belonged to the third and fourth groups, respectively. Special pellets containing 0.1% green tea catechins (Polyphenon 60, Sigma-Aldrich) in standard chow diet were prepared for mice from the first and second groups. The third and fourth groups received the same pellets without green tea catechins. All groups were fed *ad libitum*, lean mice consumed on an average 5.0 g/day, while obese mice ate only 4.3 g daily. Body weight and food intake were monitored once a week. After 4 weeks, mice were fasted for 12 h and sacrificed by cervical dislocation. Liver and small intestine (SI) were dissected, washed with saline buffer, immediately frozen in dry ice, and stored in −80°C until further use.

### Tissue RNA Extraction and cDNA Synthesis

Approximately 50 mg of liver or SI tissue were used for total RNA extraction using TriReagent according to manufacturer’s instructions (Biotech, Czech Republic). The homogenization of the samples was performed with a pestle microhomogenizer in 1.5 ml Eppendorf tube using 1 ml of TriReagent per 50 mg of tissue. RNA yields and purity were determined measuring the absorbance at 260 and 280 nm using NanoDrop ND-1000 UV-Vis Spectrophotometer (Thermo Fisher Scientific, Czech Republic). All samples had absorption ratio A260/A280 greater than 1.8. The quality of RNA was checked by agarose gel electrophoresis and the integrity by 3′:5′assay according to Nolan et al [24] (detailed protocol in Supplementary information Data S1). Ten µg of RNA were treated with DNase I (NEB) to avoid genomic DNA contamination for 20 min at 37°C, inactivated by heat (10 min at 75°C) and diluted to concentration of 0.2 µg/µl. RNA was stored at −80°C until further analyses. First strand synthesis of mRNAs was carried out using ProtoScript II reverse transcriptase and random hexamers (or oligo-dT for 3′:5′assay) following the manufacturer protocol (NEB). After initial heat denaturation of 1 µg of total RNA (65°C for 5 min), the reactions (20 µl) were incubated for 10 min at 25°C, for 50 min at 42°C and for 15 min at 75°C. For the cDNA synthesis of miRNAs and small RNAs, the reaction mixture included Stem-Loop Oligos specific for each miRNA and sno202 or specific reverse primers for sno234 and U6 [25]. First strand synthesis was carried out using ProtoScript II reverse transcriptase. After initial heat denaturation of total RNA (65°C for 5 min), the reactions (10 µl) were incubated for 30 min at 16°C, for 30 min at 42°C and for 15 min at 75°C. Obtained cDNAs were diluted (5x, 50x, and 10.000x or 100.000x for 18S analyses) prior to qPCR (details in Table S1). All cDNAs were stored at −20°C until qPCR assay.

### Primer Design, Quantitative Real-time PCR

The candidate RGs for mRNA and miRNA normalization are listed in Table 1. The primers for mRNA normalization were either obtained from PrimerBank (http://pga.mgh.harvard.edu/primerbank), designed using Primer3 software [26] or described in previous reports. For miRNA normalization the reverse primer used was universal (the sequence inserted by Stem-Loop primer [22], [25]) and the forward primers were designed manually and controlled using OligoCalc (http://www.basic.northwestern.edu/biotools/oligocalc.html) [27]. All primers were synthesized by Generi Biotech, Czech Republic. The specificity of the primers was checked by NCBI Blast tool and the reaction conditions were optimized by determining the primer concentrations. The primer sequences (with their corresponding bibliographic references), amplicon sizes, and concentrations are listed in Table 2.

**Table 1:** Description of selected candidate reference genes and genes of interest.

Gene Symbol	Gene Name	GeneBank or miRbase Accession Number	Gene Function
**Candidate reference genes for mRNA normalization**			
18S	18S Ribosomal RNA	NR_003278	Protein Synthesis
ACTB	Beta Actin	NM_007393	Cytoskeletal structural protein
B2M	Beta 2 Microglobulin	NM_009735	Beta-chain of major histocompatibility complex
GAPDH	Glyceraldehyde3-phosphate dehydrogenase	NM_008084	Glycolysis pathway enzyme
HMBS	Hydroxymethylbilane synthase	NM_013551.2	Heme biosynthetic pathway enzyme
HPRT1	Hypoxanthine phosphoribosyl transferase	NM_013556	Metabolic salvage of purines
RPlP0	Ribosomal Protein large P0	NM_007475.4	Structural constituent of ribosome
**Candidate reference genes for microRNA normalization**			
miR-16	MicroRNA 16 (5p)	MIMAT0000527	Regulation of apoptosis
miR-19a	MicroRNA 19a (3p)	MIMAT0000651	Control of endothelial cell functions
miR-122	MicroRNA 122 (5p)	MIMAT0000246	Regulation of fatty acid metabolism
miR-142	MicroRNA 142 (5p)	MIMAT0000154	Regulation of hematopoiesis
miR-143	MicroRNA 143 (3p)	MIMAT0000247	Regulation of cardiac morphogenesis
miR-186	MicroRNA 186 (5p)	MIMAT0000215	Regulation of cell senescence
miR-200a	MicroRNA 200a (3p)	MIMAT0000519	Maintenance of the epithelial phenotype
sno202	Small nucleolar RNA 202	AF357327	Modification of small nuclear RNAs
sno234	Small nucleolar RNA 234	AF357329	Modification of small nuclear RNAs
U6	U6 small nuclear RNA	NR_003027	RNA Splicing
**Genes of interest**			
NQO1	quinone oxidoreductase 1	NM_008706	Phase I metabolism
miR-221	MicroRNA 221 (3p)	MIMAT0000669	Regulation of erythropoiesis
miR-29b	MicroRNA 29b (3p)	MIMAT0000127	Neuronal maturation

**Table 2:** Description of gene-specific real-time PCR assays.

Gene Symbol	Primer sequences 5′-3′	Am (bp)	Tm (°C)	Ref	c (nM)	RNAinput(ng)	E (%)
**Candidate reference genes for mRNA normalization**							
18S	R:CTGAACGCCACTTGTCCCTC	133	85.0	P3	100	0.0025 (0.025)	99
	F:GGCCGTTCTTAGTTGGTGGAGCG				100		
ACTB	R:CCAGTTGGTAACAATGCCATGT	154	86.0	6671509a1	100	50	94
	F:GGCTGTATTCCCCTCCATCG				100		
B2M	R:GTTCGGCTTCCCATTCTCC	103	83.5	[30]	250	50	105
	F:GGTCTTTCTGGTGCTTGTCTCA				250		
GAPDH	R:TGTAGACCATGTAGTTGAGGTCA	123	86.0	6679937a1	250	50	92
	F:AGGTCGGTGTGAACGGATTTG				250		
HMBS	R:CTGGGCTCCTCTTGGAATG	168	85.0	[30]	100	50	92
	F:GATGGGCAACTGTACCTGACTG				100		
HPRT1	R:GGCCTCCCATCTCCTTCATG	167	81.5	P3	250	50	93
	F:CAGTCCCAGCGTCGTGATTA				250		
RPlP0	R:CTGGGCTCCTCTTGGAATG	136	87.5	[29]	250	50	92
	F:GATGGGCAACTGTACCTGACTG				250		
**Candidate reference genes for microRNA normalization**							
miR-16	RT:GTCTCCTCTGGTGCAGGGTCCGAGG TATTCGCACCAGAGGAGACCGCCAA			[22], [25]			
	F:ACAGCCTAGCAGCACGTAAAT	51	79.0		50	2.5 (25)	102
miR-19a	RT:GTCTCCTCTGGTGCAGGGTCCGAGGTATTCGCACCAGAGGAGACTCAGTT			[22], [25]			
	F:AGACCTCCTGTGCAAATCTATG	54	77.5		50	50	98
miR-122	RT:GTCTCCTCTGGTGCAGGGTCCGAGG TATTCGCACCAGAGGAGACCAAACA			[22], [25]			
	F:GGCTGTGGAGTGTGACAATG	50	78.5		50	2.5 (25)	100
miR-142	RT:GTCTCCTCTGGTGCAGGGTCCGAGGTATTCGCACCAGAGGAGACGTAGTG			[22], [25]			
	F:GGAGCGTGCATAAAGTAGAAAG	51	78.0		50	50 (25)	101
miR-143	RT:GTCTCCTCTGGTGCAGGGTCCGAGGTATTCGCACCAGAGGAGACGAGCTA			[22], [25]			
	F:CCGACTGAGATGAAGCACTG	49	78.5		50	50	102
miR-186	RT:GTCTCCTCTGGTGCAGGGTCCGAGGTATTCGCACCAGAGGAGACAAGCCC			[22], [25]			
	F:GGTGCGCAAAGAATTCTCCTT	52	78.5		50	50 (25)	109
miR-200a	RT:GTCTCCTCTGGTGCAGGGTCCGAGGTATTCGCACCAGAGGAGACACATCG			[22], [25]			
	F:GCGTCCTAACACTGTCTGGT	51	78.0		50	50	109
sno202	RT:GTCTCCTCTGGTGCAGGGTCCGAGGT ATTCGCACCAGAGGAGACCATCAGAT			[22], [25]			
	F:CCTGTGTACTGACTTGATGAAAG	73	78.0		50	2.5(25)	93
sno234	RT:GGATCGCCTCTCAGTGGTAG			P3			
	F:GGCTTTTGGAACTGAATCTAAGTG	63			50	25	91
U6	RT:AACGCTTCACGAATTTGCGTG			P3	250		101
	F:GCTCGCTTCGGCAGCACA	95	82.0		250	2.5	
Universal	R:GAGGTATTCGCACCAGAGGA			[22]			
**Genes of interest**							
NQO1	R:TCCTTTTCCCATCCTCGTGG	142	80.5	P3	100	50	98
	F:GTCCATTCCAGCTGACAACC				100		
miR-211	RT:GTCTCCTCTGGTGCAGGGTCCGAGGTATTCGCACCAGAGGAGACAAACCC			[22], [25]			
	F:CTGCCAGCTACATTGTCTGC	46	79.0		50	25	96
miR-29b	RT:GTCTCCTCTGGTGCAGGGTCCGAGGTATTCGCACCAGAGGAGACAACACT			[22], [25]			
	F:GCCGTTAGCACCATTTGAAATC	51	77.5		50	50	98

Am: amplicon size. Bp: number of base pairs. Tm: melting temperature. RT: retro-transcription primer. R: reverse primer. F: Forward primer. Ref: references/PrimerBankID/P3: primers were designed using Primer3 software. C: concentration of primers in qPCR reaction in nM. RNA input: respective amount of RNA in qPCR reaction in ng (in brackets: if different amount for small intestine samples were used). E: Assays efficiency.

The qPCR analyses were performed in CFX96 Touch Real-Time PCR Detection System (Bio-Rad) using SYBR Green I detection in a final volume of 20 µl. The reaction mixture consisted of components from qPCR Core kit for SYBR Green I (Eurogentec) as specified by manufacturer (details in MIQE checklist), both forward and reverse primers (final concentrations in Tab. 2), and 5 µl of diluted cDNA. The same batch of diluted cDNA (5 µl, corresponding to 50 ng of reverse transcribed RNA) was subjected to qPCR to amplify all candidate genes for mRNA normalization as well as target gene. Five µl of respective cDNAs were used for qPCR analysis of each microRNA.

The PCR reactions were initiated by the denaturation step of 10 min at 95°C, followed by 40 cycles of amplification, which were performed according to the following thermo cycling profiles: denaturation for 10 sec at 95°C and annealing and extension for 40 sec at 60°C. Fluorescence data were acquired during the last step. Dissociation protocol with a gradient (0.5°C every 30 s) from 65°C to 95°C was used to investigate the specificity of the qPCR reaction and presence of primer dimers. Gene-specific amplification was confirmed by a single peak in the melting curve analysis (Fig. S1). The size of all amplicons was confirmed by 2% agarose gel electrophoresis stained with SYBR Safe DNA gel stain (Invitrogen). The sample maximization method criterion was used to establish the run layout.

The absence of contamination from either genomic DNA amplification or primers dimers formation was ensured using two types of controls, the first one without reverse transcriptase (no-RT control, one for each RNA), and the second one with no DNA template (NTC control, one for each primer pair). All qPCRs were run in duplicates, the average standard deviation within duplicates of all samples studied was 0.15 cycles. qPCR efficiencies in the exponential phase were calculated for each primer pair by standard curves (5point 5-fold dilution series of pooled cDNA), the mean quantification cycle (Cq) values for each serial dilution were plotted against the logarithm of the cDNA dilution factor and calculated according to the equation E = 10[-1/slope] [15]. The amplification efficiencies for all evaluated candidate RGs ranged from 91% to 109% (Table 2). The parameters derived from the qPCR analysis required by the MIQE guidelines are listed in Table S1.

### Data Analysis

To select a suitable RG, the stability of mRNA expression of each RG was statistically analyzed with three freely available Microsoft Excel-based software packages: geNorm [http://medgen.ugent.be/~jvdesomp/genorm/] (Vandesompele), NormFinder [http://moma.dk/norminder-software], and BestKeeper [http://gene-quantification.com/bestkeeper.html]. For geNorm and NormFinder, the raw Cq values were transformed into relative quantities - the required data input format. The maximum expression level (the lowest Cq value) of each gene was used as a control and was set to a value of 1. Relative expression levels were then calculated from Cq values using the formula: 2^⁁^(−ΔCq), in which ΔCq represents each corresponding Cq value – minimum Cq value [28]. The obtained data were further analyzed with geNorm and NormFinder. BestKeeper analyses were based on untransformed Cq values. For consensus ranking of all candidate RGs geometric mean of ranks from these three analyses was calculated.

The gene expression simulation was performed using data from the three biological replicates in each group. Relative and normalized fold expression values were calculated manually in Microsoft Excel from Cq values imported from CFX Manager (Bio-Rad). Expression data were imported to GraphPad Prism v6 (GraphPad Software, Inc.) and the statistical significances of the results were compared and analyzed with two way analysis of variance (ANOVA) and multiple comparisons using uncorrected Fisher’s LSD test. Differences were scored as statistically significant at the P<0.05.

## Results

Newborn mice were injected with MSG and kept for 7 months until obesity, diabetes and metabolic syndrome developed. Hyperleptinemia and hyperinsulinemia were confirmed by enzyme-linked immunosorbent assays (data not shown). Afterwards, mice were fed for one month with green tea catechins-supplemented diet and then the liver tissue and SI were dissected. With the aim to analyze and compare mRNA and miRNA profile in different groups of animals, the RGs for this experimental set up were searched. These RGs served for normalized quantification of selected mRNA and miRNA in liver and SI and their comparison in four groups of mice: I/lean mice with green tea catechins-enriched diet, II/obese mice with green tea catechins-enriched diet, III/lean mice with standard diet and IV/obese mice with standard diet.

### Expression Profiles of Reference Genes

The selection of candidate RGs for mRNA and for miRNA was performed separately, using two different sets of genes (Table 1). In the case of the RGs for mRNA normalization, the evaluated genes were beta-2 microglobulin (B2M), beta-actin (ACTB), glyceraldehyd-3-phosphate dehydrogenase (GAPDH), hypoxanthine phosphoribosyltransferase 1 (HPRT1), large subunit of ribosomal protein P0 (RPlP0), hydroxymethylbilane synthase (HMBS), and ribosomal RNA 18S (18S). In the case of miRNA normalization, the evaluated candidate RGs included miR-16, miR-19a, miR-122, miR-142, miR-143, miR-186, miR-200a, small nucleolar RNA 202 and 234 (sno202, sno234) and small nuclear RNA U6 (U6). All candidate RGs for mRNA and miRNA normalization were selected considering 1) that they belong to different functional classes in order to reduce the chance of co-regulation of their genes, 2) their common use as endogenous controls and 3) their relative quantities in liver and/or SI [29]–[33].

**Figure 1:**
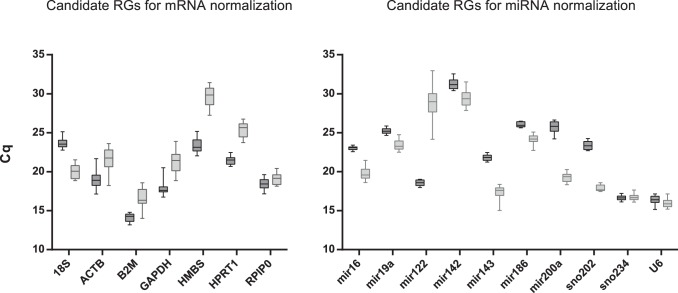
Range of quantification cycle (Cq) values of the candidate reference genes. Boxplot of quantification cycles (Cq) values for each reference gene for mRNA and miRNA normalization in all liver (dark grey box) and small intestine (light grey box) samples was assessed (n = 12). The box indicates the 25% and 75% percentiles. Whiskers represent the maximum and minimum values. The median is depicted by the line across the box.

A qPCR assay based on SYBR Green I dye detection was carried out to examine the stability of candidate genes’ expression. The raw Cq values were extracted from the CFX Manager (Bio-Rad) and represented by box-and-whiskers plots (Fig. 1). In the case of mRNA candidate RGs, 18S was the most abundantly expressed gene in our study due to its different nature, therefore the input cDNA was diluted to circumvent the question whether 18S represents the best reference control when normalizing a low abundance target gene. The dilution of cDNA prior to qPCR was performed within the linear range of the amplification curve generated for 18S. Apart from 18S, B2M was the most highly expressed gene in both tissues. All genes showed higher level of expression in liver samples. The most apparent difference between liver and SI (more than 6 cycles) was observed for HMBS. In the case of miRNA quantification, the most abundantly expressed and the least expressed genes in both tissues were U6 and miR-142, respectively.

### Rank of Reference Genes According to their Expression Stability

#### GeNorm analysis

**Figure 2:**
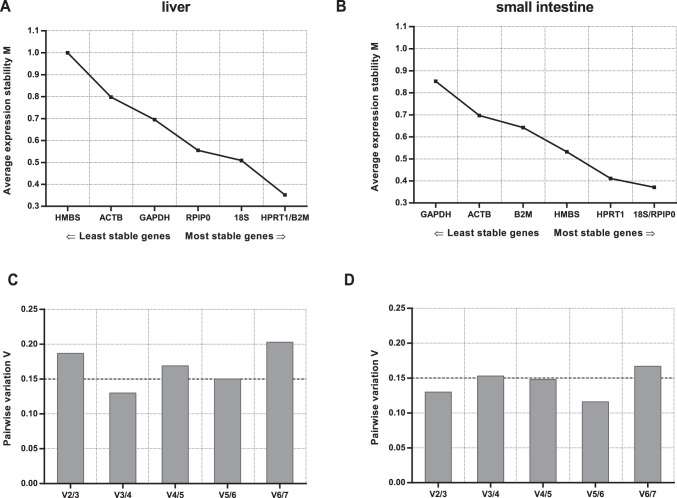
geNorm analysis of candidate reference genes for mRNA normalization. Genes presented on the x-axis in the order of increasing stability (M value on y-axis) for liver and small intestine (A, B). Determination of the optimal number of control genes for normalization in liver and small intestine (C, D).

GeNorm [18] provides a ranking of the tested genes based on their expression stability measure (M), which is average pairwise variation of a particular gene with all other control genes. M values lower than 0.5 are typically observed for stably expressed genes in relatively homogenous sample panels [34]. Since the levels of expression of tested RGs in liver and SI tissue samples differ, the results are analyzed separately. The ranking order for mRNA normalization according to the M value is shown in Figure 2. The most stably expressed genes in liver tissue were HPRT1 and B2M (with the M value of 0.352), while 18S and RPlP0 with the M value of 0.371 were the most stably expressed genes in SI (Table 3).

**Table 3:** Ranking of the candidate mRNA RGs according to their stability value using geNorm, NormFinder, and BestKeeper algorithms.

	liver							small intestine						
	geNorm		NormFinder		Best keeper			geNorm		NormFinder		Best keeper		
Gene name	Stability value*	Rank	Stability value*	Rank	SD#	Rank	Consensus^»^	Stability value*	Rank	Stability value*	Rank	SD#	Rank	Consensus^»^
**18S**	0.509	3	0.179	2	0.55	4	**3**	0.371	1	0.197	2	0.72	2	**2**
**ACTB**	0.798	6	0.654	6	0.96	6	**6**	0.697	6	0.528	6	1.14	7	**6**
**B2M**	0.352	1	0.307	3	0.38	1	**1**	0.642	5	0.481	5	1.00	5	**5**
**GAPDH**	0.695	5	0.972	7	1.03	7	**6**	0.852	7	0.798	7	1.10	6	**7**
**HMBS**	1.000	7	0.544	5	0.82	5	**6**	0.532	4	0.315	4	0.97	4	**4**
**HPRT1**	0.352	1	0.122	1	0.39	2	**1**	0.411	3	0.180	1	0.82	3	**2**
**RPlP0**	0.555	4	0.385	4	0.53	3	**4**	0.371	1	0.249	3	0.59	1	**1**
**Best Gene**	B2M/HPRT1		HPRT1		B2M			18S/RPlP0		HPRT1		RPlP0		
**Best combination**	HPRT1/B2M/18S							18S/RPlP0/HPRT1						

High expression stability is indicated by low stability value.

^#^ SD; standard deviation of the coefficient of variance, SD>1 can be considered inconsistent.

»Consensus rank calculated as geometric mean of all rankings.

Another important analysis performed using geNorm was the determination of optimal number of RGs,of the effect of adding an extra gene in the analysis, by calculating the pairwise variation (Vn/Vn+1) between two sequential candidate genes. As proposed by Vandesompele et al, geNorm defines a pairwise variation of 0.15 as the cut-off value, below which the inclusion of an additional RG is unnecessary [18]. Here the V3/4 value for liver was 0.130 and V2/3 value for SI was 0.130 suggesting three or two RGs as optimal, respectively (Fig. 2).

**Figure 3:**
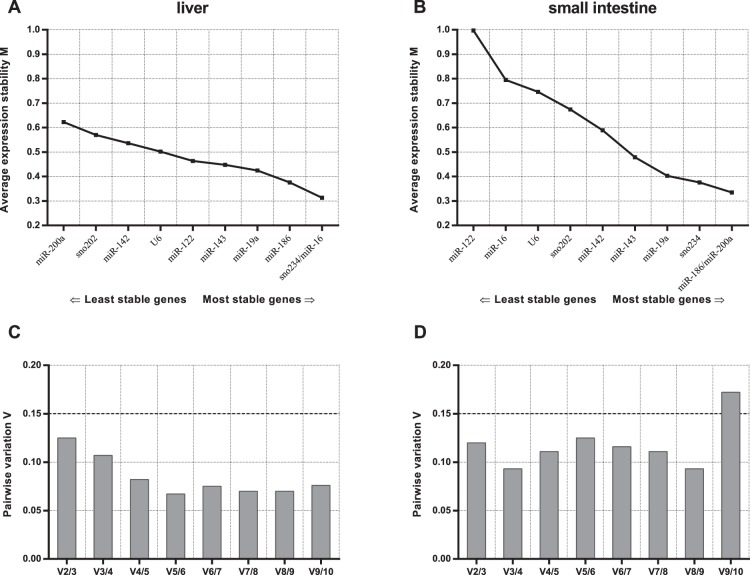
geNorm analysis of candidate reference genes for miRNA normalization. Genes presented on the x-axis in the order of increasing stability (M value on y-axis) for liver and small intestine (A, B). Determination of the optimal number of control genes for normalization in liver and small intestine (C, D).

In the case of miRNA normalization, the best RGs according to geNorm analysis are miR-16 and sno234 for liver tissue and miR-186 and miR-200a for SI. This demonstrates the importance of validating suitable RGs in a tissue-specific context, because miR-200a was assigned as the most unstable RG in the liver tissue. Based on the pairwise variation calculated for both tissues, the use of two genes is recommended (Fig. 3).

### NormFinder Analysis

NormFinder [19] employs a model-based approach that, in addition to the overall expression level variation, also takes into account the intra- and intergroup variations of the candidate normalization genes to evaluate the expression stability. Independently on groups, the top-ranked RG was HPRT1 for both tissues (Table 3). Using NormFinder, we also employed various grouping, i.e. “treatment” grouping: all control samples (both lean and obese mice) versus all catechin treated samples or “obesity” grouping: lean versus obese mice (treated or untreated) or four separate groups (untreated and treated lean or obese mice), for calculations of intra- and intergroup variations. For all possible grouping applied, 18S/HPRT1 were the best RG combination in liver sample set. In SI 18S/HMBS and RPlP0/HMBS were the best combination in treatment grouping and in obesity grouping, respectively. When all four groups were analyzed separately, RPlP0/HPRT1 were assigned as the best combination in SI (Data S2).

For miRNA normalization, the top-ranked RGs were miR-122 and sno234 for liver and SI, respectively (Table 4). When various grouping was applied, the best combination for liver tissue was miR-122 and miR-16. In SI sno234 and miR-200a were the best RGs independently on grouping (Data S2).

**Table 4:** Ranking of the candidate miRNA RGs according to their stability value using geNorm, NormFinder, and BestKeeper algorithms.

	liver							small intestine						
	geNorm		NormFinder		Best keeper			geNorm		NormFinder		Best keeper		
Gene name	Stability value*	Rank	Stability value*	Rank	SD#	Rank	Consensus^»^	Stability value*	Rank	Stability value*	Rank	SD#	Rank	Consensus^»^
miR-16	0.313	1	0,195	2	0,18	1	**1**	0.795	9	0,595	9	0,69	8	**9**
miR-19a	0.425	4	0,274	5	0,31	4	**5**	0.403	4	0,338	4	0,51	6	**5**
miR-122	0.464	6	0,164	1	0,34	5	**3**	0.997	10	1,183	10	1,55	10	**10**
miR-142	0.536	8	0,372	8	0,54	9	**8**	0.589	6	0,541	8	0,84	9	**8**
miR-143	0.448	5	0,209	3	0,34	6	**6**	0.479	5	0,486	6	0,65	7	**6**
miR-186	0.376	3	0,220	4	0,28	3	**4**	0.335	1	0,247	3	0,44	3	**2**
miR-200a	0.623	10	0,518	10	0,62	10	**10**	0.335	1	0,227	2	0,44	4	**2**
sno202	0.570	9	0,462	9	0,49	8	**9**	0.674	7	0,383	5	0,34	2	**4**
sno234	0.313	1	0,275	6	0,24	2	**2**	0.376	3	0,164	1	0,32	1	**1**
U6	0.502	7	0,318	7	0,41	7	**7**	0.746	8	0,499	7	0,44	5	**7**
**Best Gene**	miR-16/sno234		miR-122		miR-16			miR-186/miR-200a		sno234		sno234		
**Best** **combination**	mir-16/sno234 (miR-122)							sno234/miR-186 (miR-200a)						

High expression stability is indicated by low stability value.

^#^ SD; standard deviation of the coefficient of variance, SD>1 can be considered inconsistent.

»Consensus rank calculated as geometric mean of all rankings.

### BestKeeper Analysis

BestKeeper [20] calculates the coefficient of variance and standard deviation (SD) of the raw Cq values and establishes the BestKeeper index, which is the geometric mean of the Cq values of all suitable candidate RGs. The most stable RGs were identified based on having the lowest SD and the highest correlation to the BestKeeper (Data S3). Genes with SD greater than 1 were assumed to be inconsistent. BestKeeper analyses indicated that B2M, HPRT1 and RPlP0 were the most stably expressed genes in the liver (Table 3). The RPLP0, 18S and HPRT1 were the most stable RGs in the SI.

The BestKeeper analysis of miRNA candidate genes ranked mir-16 as the best RG, followed by sno234 and miR-186 for liver tissue and sno234 followed by sno202 and miR-186 for SI (Table 4, Data S3).

The comparison of summarized data in Table 3 and 4 shows that the results provided by geNorm, NormFinder and BestKeeper displayed slight differences in the ranking of the genes, but some consensus can be obtained. The most suitable combination of RGs in the liver tissue seems to be B2M, HPRT1, 18S for mRNA normalization and miR-16 and sno234 for miRNA normalization. The best combination of RGs for analyses in SI would be RPlP0, 18S, HPRT1 and miR-186 and sno234 for mRNA and miRNA normalization, respectively. The least stable genes in both tissues were GAPDH and ACTB indicating that these commonly used RGs are unsuitable for normalization in MSG mouse liver and SI. Commonly used U6 gene for miRNA normalization was ranked in the middle of the candidate RGs and therefore it seems rather inappropriate to use U6 as a single RG without proper validation prior to expression studies.

### Reference Gene Validation

**Figure 4:**
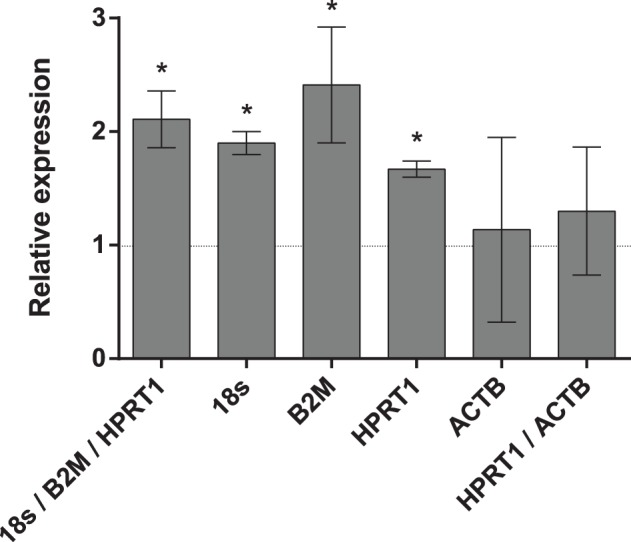
Effects of different normalization approaches on the expression of NQO1. Fold expression changes of NQO1 gene in liver after green tea catechins treatment was normalized to individual or combined reference genes. Error bars show the standard error calculated from three biological replicates. Stars indicate the significant (P<0.05) difference identified by uncorrected Fisher’s LSD test in multiple comparisons after two-way ANOVA.

The use of different RGs to calculate relative expression data could have a significant influence on the final normalized results. To demonstrate the effect of different RGs on the outcome of a practical experiment, the relative expression patterns of NAD(P)H:quinone oxidoreductase 1 (NQO1) gene were analyzed. The NQO1 gene was significantly overexpressed in the livers of lean mice after catechin feeding when the most stable references 18S, B2M and HPRT1 selected by all programs were used as the combination or as single internal controls. When the least stable reference gene ACTB was used for normalization, either as a single internal control or in combination with the best reference gene HPRT1, no increase of NQO1 was observed (Fig. 4).

**Figure 5:**
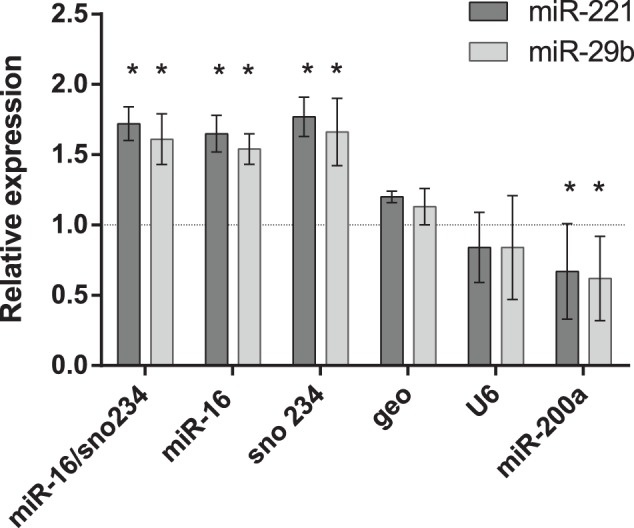
Effects of different normalization approaches on the expression of miR-221 and miR-29b. Fold expression changes of miR-221 (dark grey columns) and miR-29b (light grey columns) in MSG-obese mice were normalized to individual or combined reference genes. Error bars show the standard error calculated from three biological replicates. Stars indicate the significant (P<0.05) difference identified by uncorrected Fisher’s LSD test in multiple comparisons after two-way ANOVA.

Previous reports have shown overexpression of miR-221 in obese mice and miR-29b in diabetic animals [35], [36]. In order to evaluate the RGs analyzed in this report, we studied the liver expression of miR-221 and miR-29b using the same experimental design. According to our expectations, the increase in both miRNAs was observed, when the two best RGs (miR-16 and sno234) were used as normalizer both in combination (geometric mean) and as single RGs (Fig. 5). If the most often used RG for miRNA studies U6 or geometric mean of all studied RGs were used, the difference in miR-221 and miR-29b expressions would be overlooked.

## Discussion

In all experiments, the purpose of normalization is to remove the sampling differences (such as RNA quantity and quality) in order to identify real gene-specific variations. For proper internal control genes, this variation should be minimal or none. The identification of suitable endogenous control genes is an important initial step in expression analysis since usage of an unstable gene for normalization could result in misleading conclusions.

Although MSG mice have been frequently used for obesity study [7], the RGs have not been properly validated in this model so far. Liu et al used beta-actin in MSG-treated rats when long term effects of fenofibrate treatment on pancreatic beta cells were studied [37]. Shen et al reported mRNA normalization in skeletal muscle to GAPDH only when treated obesity using pentamethylquercetin [38]. Alponti et al studied the effect of MSG on the expression of aminopeptidase using a GAPDH as a normalizer [39]. Alarcon-Aguilar studied association of obesity and chronic inflammation using RPlP0 as a single RG for normalization in liver and visceral adipose tissue [29], [40]. Yamazaki examined MSG mice liver after SRT1720 (sirtuin 1 activator) treatment, and used 18S as a single RG [10]. Although the latter studies chose stably expressed RGs, more appropriate approach would be to test more RGs for stability in a given experimental set up prior to normalization. Ribosomal RNAs, such as 18S, 28S, 5S and 16S, are very common control genes in gene expression studies, as they are abundant and necessary in any cell type. Therefore, in order to have representative of the rRNA category, the 18S rRNA was included in our set of candidate genes. Since the 18S rRNA is not mRNA and the degradation machinery may not affect rRNA and mRNA in the same manner, some authors did not consider it as a real internal control for gene expression studies [41]. However, in our study the 18S rRNA showed to be one of the best references and we could recommend its use in a combination with at least one other RG.

Based on combination analysis of the seven tested RGs for mRNA quantification using three statistical programs, 18S/HPRT1 and 18S/RPlP0 were selected as the best RGs for liver and small intestine in the given experimental set up. Ideally, these RGs can be combined with one more gene, B2M and HPRT1 for liver and SI, respectively. The use of an increased number of RGs in the normalization can improve the reliability of a study, but it is time-consuming and more expensive and thus, a trade-off between the gain in accuracy, costs and time involved needs to be carefully balanced. In the analysis of our data, the determination of the pairwise variation of two sequential normalization factors (Vn/n+1) using the geNorm software indicated that the minimum number of RGs to be included in the normalization of liver and SI sample set was three and two, respectively. However, we propose several RGs should be tested prior to normalization in the given experimental set up and used treatment (*i.e.* 18S, HPRT1, B2M and RPlP0 or 18S, HPRT1, HMBS and RPlP0 for gene expression analysis in liver and SI, respectively). Usage of ACTB and GAPDH as normalizers for MSG mice is not recommended as all used programs ranked these genes as the least stable. Moreover, bioinformatics data show that ACTB and GAPDH have many pseudogenes in the human and mouse genomes, which may affect the fidelity of these genes as references for qPCR [42].

In the present study, the selected RGs were used for comparison of hepatic expression of certain enzyme in mice receiving catechins-enriched diet and in mice on standard chow diet. NQO1, important drug-metabolizing and detoxifying enzyme, was chosen for this purpose as previous experiments showed that green tea extract enhanced the expression of NQO1 in human cell lines [43]. Using 18S, B2M and HPRT1 genes for normalization, significant overexpression of the NQO1 gene in the liver of catechin-treated mice was found. These finding is in agreement with results of some other studies indicating that green tea catechins may exert a significant effect on drug metabolism and in turn may affect the ability of the organism to detoxify eobiotics and xenobiotics (reviewed in [6]).

MiRNAs have been recognized as critical factors in gene regulation of either physiological or pathological processes. To date, more than 30,000 mature miRNAs from 206 species are registered in the miRSanger Base (Release 20, June 2013) (http://www.mirbase.org) [44]. Emerging evidence suggests that miRNAs play a key role in the pathological development of obesity by affecting adipocyte differentiation. The inverse pattern of miRNA expression observed in differentiating adipocytes and in mature adipocytes indicates that obesity leads to a loss of miRNA that characterize fully differentiated and metabolically active adipocytes [45], [46]. Investigation of these tiny molecules and their genetic targets may potentially identify new pathways involved in the metabolic disease processes, improving our understanding of metabolic disorders and influence future approaches to the treatment of obesity. Importantly, recent studies have shown that many phytochemicals, including green tea catechins, could alter the expression of specific miRNAs [47], [48].

Since miRNA regulates gene expression by a ‘fine-tuning’ mechanism, the study of the participation of miRNA in specific physiological or pathological experimental situations depends on a reliable and accurate technique for measuring their expression levels. However, relative quantification of miRNA is complicated by the fact that cDNA for each sample is prepared using miRNA-specific primers, thereby introducing additional non-biological variation not involved in the synthesis of cDNA from mRNA when using random or oligo-dT primers. Therefore, the reliability of ten candidate RGs for normalization of miRNA quantification was systemically evaluated in present study.

As previously reported, a proportion of small RNAs may exhibit tissue-specific and developmental regulation [49]. In our experiment, the expression of sno202 in the liver was highly unstable compared to the expression of one of the best RGs sno234, which may indicate some obesity-related regulation of sno202. Recently, Vandesompele and coworkers have described an alternative approach to normalize qPCR experiments. They circumvent the difficulty in identifying the reliable RGs by using the global mean miRNA expression value as a normalization method [13]. However, when the global geometric mean of all miRNA RGs evaluated in this study was applied, the expression difference got lost, possibly given by the small number of microRNAs studied and considerable influence of the unstable miRNAs (*i.e*. miR-200a) in the sample set. The use of unsuitable references can lead to over- or underestimation of relative transcript abundance. These results reinforce the importance of validating RGs prior to experimental applications.

In the present study, the RGs selected for miRNA profiling were tested in obese (MSG-treated) and lean mice with miR-221 and miR-29b as target miRNAs. In agreement with previous data [35], [36], significant increase in both miRNAs was observed in MSG-obese mice in comparison to lean ones, when the two best RGs (miR-16 and sno234) were used as normalizer. As it is evident from our results, inappropriate use of RGs can significantly alter the results of target miRNA quantification.

In conclusion, we present the first experimentally validated comparison of RGs for the normalization of mRNA and microRNA qPCR expression data in MSG mouse model of obesity. The combined use of B2M/18S/HPRT1 and miR-16/sno234 for normalization was validated as the optimal RGs for mRNA and microRNA expression data from liver, respectively. The combined use of 18S/RPlP0/HPRT1 and sno234/miR-186 is recommended for normalization in the samples of small intestine. These reference genes will be used for further study of green tea catechins action in obese mice.

## Supporting Information

Figure S1 Representative amplification and melt-curve profiles of Real-Time qPCR assays.
(PDF)

Table S1 Checklist MIQE.
(PDF)

Data S1 Description of 3′:5′assay.
(PDF)

Data S2 Stability values of the candidate reference genes for mRNA and miRNA normalization calculated by NormFinder with different grouping applied.
(PDF)

Data S3 BestKeeper analysis.
(PDF)
