# Supplementary material for: Reference Genes for Real-Time PCR Quantification of Messenger RNAs and MicroRNAs in Mouse Model of Obesity
Source: PLoS One. 2014 Jan 17;9(1):e86033. doi: 10.1371/journal.pone.0086033 (PMC3895018; doi:10.1371/journal.pone.0086033)

Figure S1

Representative amplification and melt-curve profiles of Real-Time qPCR assays

Candidate reference genes for mRNA normalization

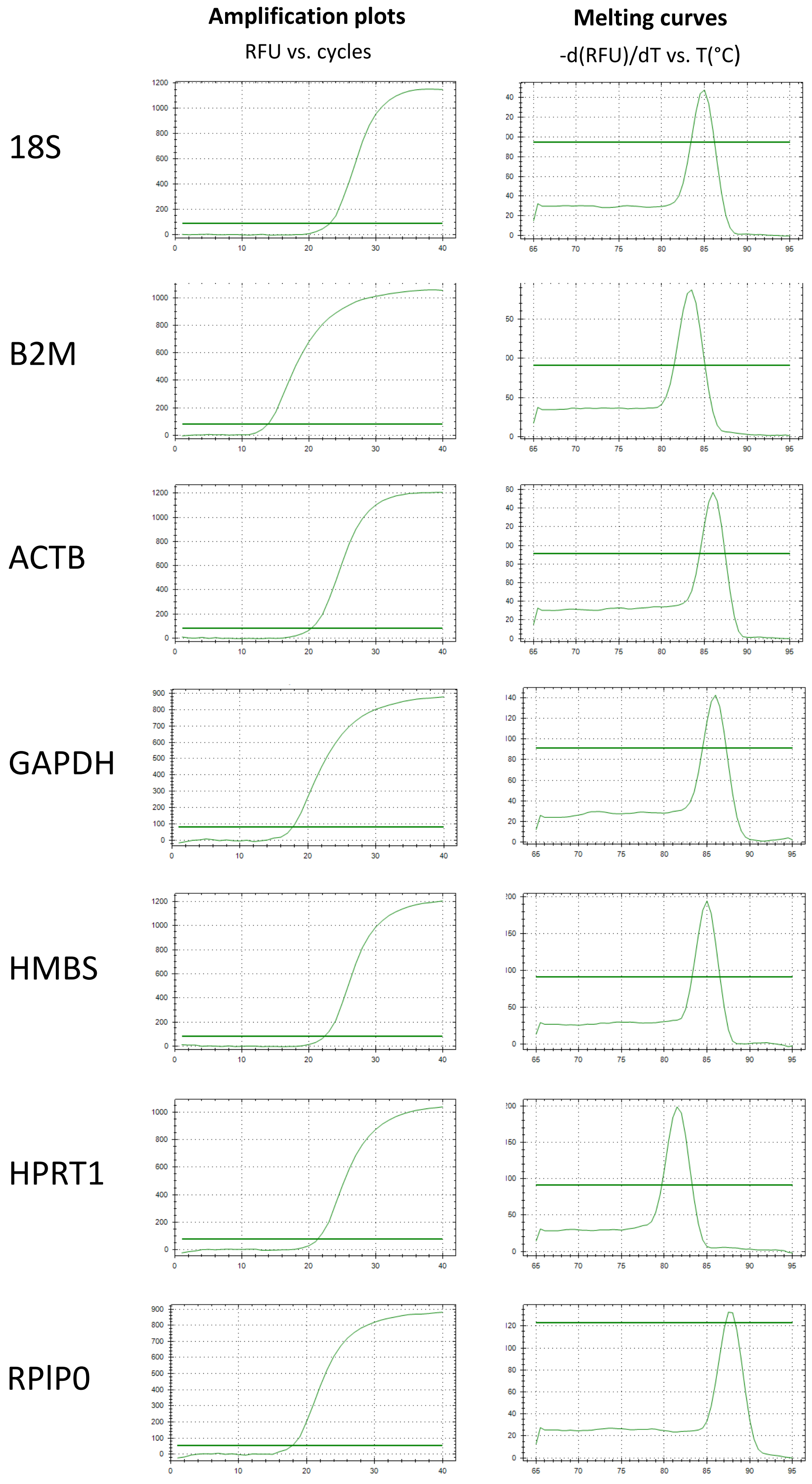

Representative amplification and melt-curve profiles of Real-Time qPCR assays

Candidate reference genes for microRNA normalization

Amplification plots
RFU vs. cycles

Melting curves
-d(RFU)/dT vs. T(°C)

miR-16

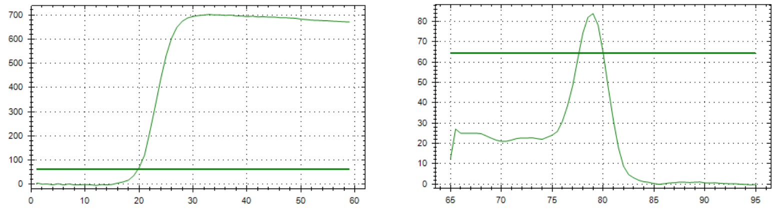

miR-19a

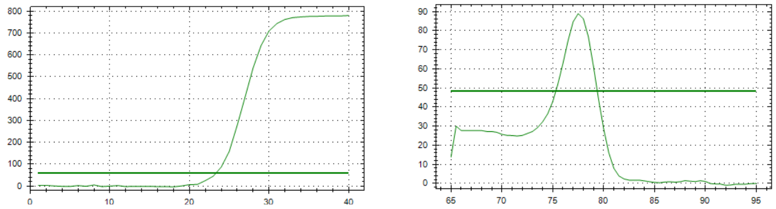

miR-122

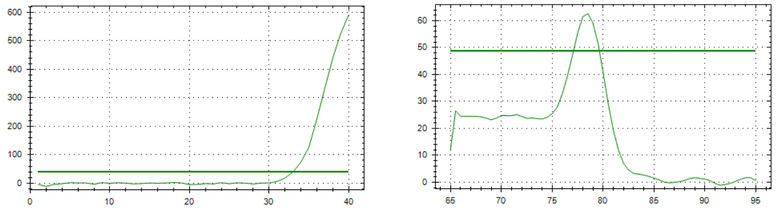

miR-142

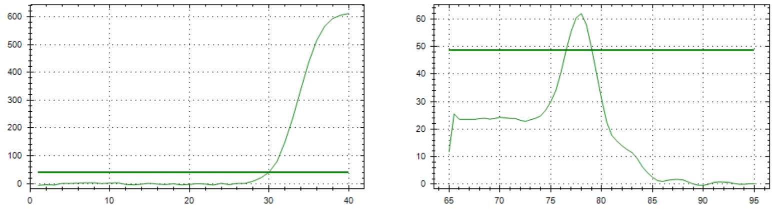

miR-143

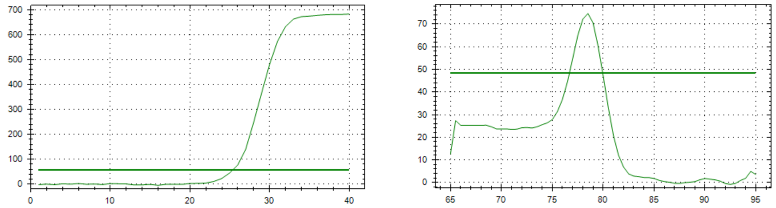

miR-186

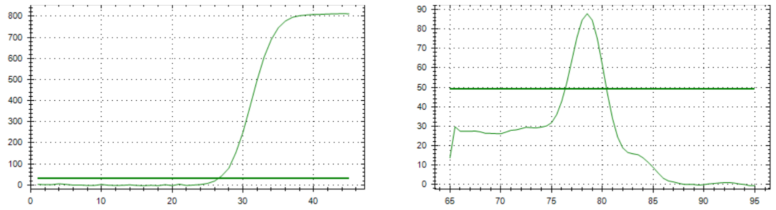

miR-200a

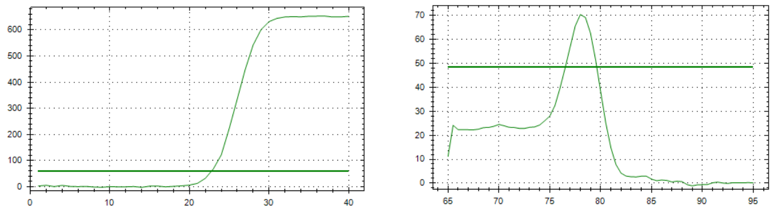

Representative amplification and melt-curve profiles of Real-Time qPCR assays

Candidate reference genes for microRNA normalization

Amplification plots — RFU vs. cycles

Melting curves — -d(RFU)/dT vs. T(°C)

sno202

sno234

U6

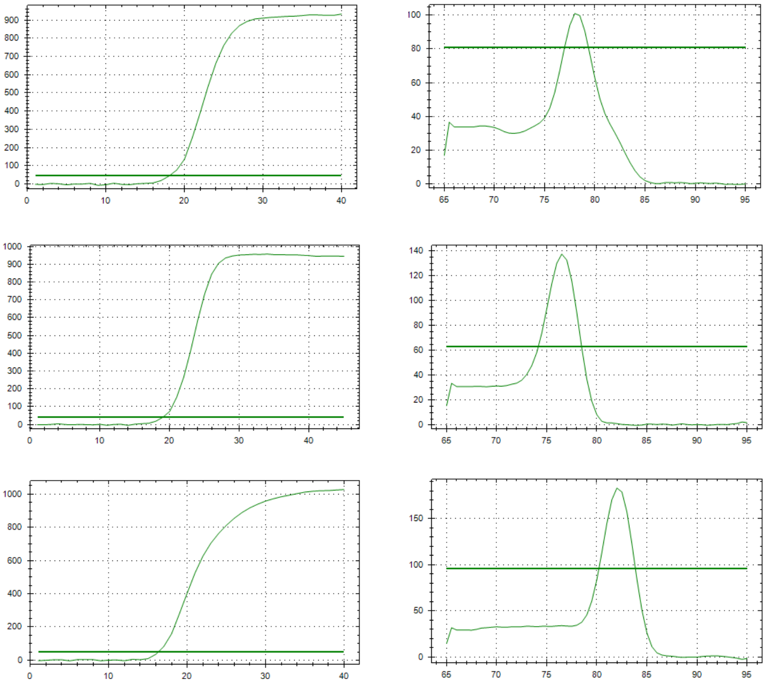

Genes of interest

Amplification plots — RFU vs. cycles

Melting curves — -d(RFU)/dT vs. T(°C)

NQ01

miR-221

miR-29b

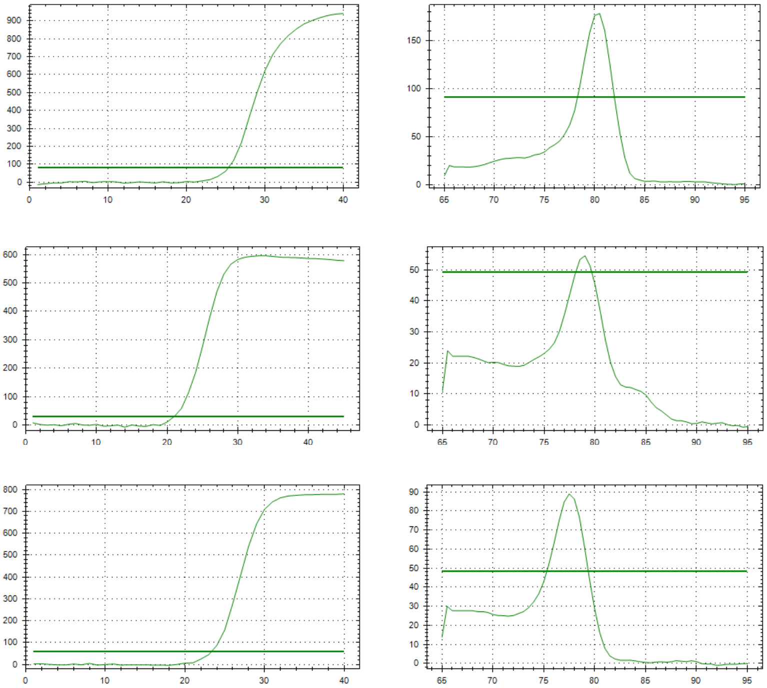

Supplement: Figure S1 — Representative amplification and melt-curve profiles of Real-Time qPCR assays. (PDF) [file pone.0086033.s001.pdf]
