# Supplementary material for: Reference Genes for Real-Time PCR Quantification of Messenger RNAs and MicroRNAs in Mouse Model of Obesity
Source: PLoS One. 2014 Jan 17;9(1):e86033. doi: 10.1371/journal.pone.0086033 (PMC3895018; doi:10.1371/journal.pone.0086033)
Supplement: Table S1 — Checklist MIQE. (PDF) [file pone.0086033.s002.pdf]

Table S1

Checklist MIQE

Item to check	Importance	
**Experimental design**		
Definition of experimental and control groups	E	I/ normal mice with green tea catechins-enriched diet (Polyfenon 60, Sigma-Aldrich), II/ obese mice with green tea catechins-enriched diet, III/ normal mice with standard diet (control group) and IV/ obese mice with standard diet.
Number within each group	E	N=3
Assay carried out by the core or investigator's laboratory?	D	investigator´s laboratory
Acknowledgment of authors' contributions	D	Conceived and designed the experiments: PM BS LS. Performed the experiments: PM HB VH IB BS. Analyzed the data: PM. Contributed reagents/materials/analysis tools: IB BS LS. Wrote the paper: PM IB LS. All authors read and approved the final manuscript.
**Sample**		
Description	E	Fresh livers and small intestines from all mice
Volume/mass of sample processed	D	50mg of tissue
Microdissection or macrodissection	E	Macrodisssection
Processing procedure	E	Mice were anesthetized with ether and sacrificed by cervical dislocation. Liver and small intestine were disected.
If frozen, how and how quickly?	E	Samples were washed with saline buffer after disection and immediately frozen in powdered dry ice and stored in -80 freezer
If fixed, with what and how quickly?	E	Not fixed
Sample storage conditions and duration	E	Samples were held at -80°C for up to one motnh before RNA isolation
**Nucleic acid extraction**		
Procedure and/or instrumentation	E	Homogenization of samples was performed using pestle microhomogenizator in 1.5 ml Eppendorf tube using 1ml of TriReagent per 50mg of tissue (liver or small intestine). Total RNA was extracted using the TriReagent (Biotech) following manufacture's protocol. The purified RNA was dissolved in 100 ul DEPC-Treated Water (0.01%DEPC in HPLC water, autoclaved) and stored at -80°C.
Name of kit and details of any modifications	E	TriReagent (Biotech, TR-118). We exactly followed manufacture's protocol.
Source of additional reagents used	D	Chloroform (Chemapol); 2-propanol (Sigma-Aldrich); absolute Ethanol (Sigma-Aldrich); DEPC (Sigma-Aldrich)
Details of DNase or RNase treatment	E	10µg of RNA was treated with 2U of Dnase I (Biotech) in 30µl final volume reaction. Digestion of DNA was achieved with 30 minutes incubation at 37°C. 1.5 µl of 100mM EDTA was added an the reaction was incubated for 10min at 65°C for inactivation and diluted up to 50µl with DEPC-treated water.
Contamination assessment (DNA or RNA)	E	Reverse transcription controls (without enzyme) were performed in order to assess the absece of DNA in the RNA sample - done for all samples in the RT step using random hexamers (not repeated in all microRNA RT steps), samples were processed along with the normal RT samples, except that no reverse transcriptase was added to the reaction mixture.
Nucleic acid quantification	E	RNA concentration was determined by measuring the absorbance at 260 nm UV light
Instrument and method	E	NanoDrop ND-1000 UV-Vis Spectrophotometer (Thermo Scientific)
Purity (A260/A280)	D	The absorbance ratio 260/280 of all samples proceed was higher than 1.9
Yield	D	-
RNA integrity: method/instrument	E	The RNA integrity was assessed by visual inspection on 2% agarose gel visualized by SYBR Safe Staining
RIN/RQI or Cq of 3' and 5' transcripts	E	3´: 5´ GAPDH assay -detailed description in data S1
Electrophoresis traces	D	-
Inhibition testing (Cq dilutions, spike, or other)	E	The standard curve has been considered sufficient to rule out the presence of inhibitors of reverse-transcription activity or PCR, also taking into account the high quality of starting RNAs.

Reverse transcription		
Complete reaction conditions	E	REVERSE TRANSCRIPTION OF mRNA: 5 ul of RNA (from the DNase I treatment reaction mixture=1ug of RNA) was firstly incubated with 1 ul random hexamers 0.1mM, 2 ul dNTP Mix 5 mM and 4 ul H_2O . This mixture was heated to 65°C for 5 min and then incubated on ice for at least 1 min for primer annealing and spin down shortly. Finally, the sample was mixed with 4 ul 5X ProtoScript II RT Reaction Buffer, 2 ul 10x DTT, 1 ul RNase Inhibitor 40 U/ul incubated for 2 min at 42°C and 1 ul ProtoScript II 200 U/ul was added and mixed by pipetting. Reactions were incubated in a PCR MJ Mini (Bio-Rad) at 25 °C for 10 min, 42 °C for 50 min and 75°C for 15 min to stop the reaction. The same reaction mixture except for Protoscript II was used for the No-RT control.
		REVERSE TRANSCRIPTION OF microRNA: 1.25 or 2.5 ul of RNA (from the DNase I treatment reaction mixture=250 or 500ng of RNA) was firstly incubated with 0.5 ul specific primers 5nM, 1 ul dNTP Mix 5 mM and 3.25 or 2 ul H_2O (depending on RNA input). This mixture was heated to 65°C for 5 min and then incubated on ice for at least 1 min for primer annealing and spin down shortly. Finally, the sample was mixed with 2 ul 5x ProtoScript II RT Reaction Buffer, 1 ul 10x DTT, 0.5 ul RNase Inhibitor 40 U/ul and 0.5 ul ProtoScript II 200 U/ul and mixed by pipetting. Reactions were incubated in a PCR MJ Mini (Bio-Rad) at 16 °C for 10 min, 42 °C for 50 min and 75°C for 15 min to stop the reaction.
Amount of RNA and reaction volume	E	Amount of RNA for mRNA: **1ug**; Reaction volume: **20 ul**; for miRNA: **0.5/0.25ug**; Reaction volume **10ul** (specified in Table 2)
Priming oligonucleotide (if using GSP) and concentration	E	random hexamers: 100 uM; gene specific primers (U6, sno234) and Stem Loop-Oligos for microRNAs and sno202: 5uM; oligo(dT)18 (for 3´:5´assay): 50uM
Reverse transcriptase and concentration	E	ProtoScript II (NEB) in final concentration:10 U/ul
Temperature and time	E	mRNA: 65°C for 5min, 25°C for 10 min, 42°C for 50 min and 75°C for 15 min
		microRNA: 65°C for 5min, 16°C for 30 min, 42°C for 30 min and 75°C for 15 min
Manufacturer of reagents and catalogue numbers	D	DNase I (Biotech, M0303S); dNTP Mix with dTTP (Eurogentec, NU-0010-10); ProtoScript II (NEB, Cat. M0368L); RNase Inhibitor (Biotech, M0307S)
Cqs with and without reverse transcription	D	-
Storage conditions of cDNA	D	-20°C
**qPCR target information**		
Gene symbol	E	Table 1
Sequence accession number	E	Table 1
Location of amplicon	D	-
Amplicon length	E	Table 2
In silico specificity screen (BLAST, and so on)	E	Described in text
Pseudogenes, retropseudogenes, or other homologs?	D	-
Sequence alignment	D	-
Secondary structure analysis of amplicon	D	-
Location of each primer by exon or intron (if applicable)	E	B2M(forward:1st exon,reverse:2nd exon); ACTB(f:2nd,r:3rd); GAPDH(f:1st,r:3rd);HMBS(f:11th,r:14th); HPRT1(f:1st, r:3rd); RPlP0 (f:6th,r:7th); NQO1 (f:6th,r:6th)
What splice variants are targeted?	E	-

**qPCR oligonucleotides**		
Primer sequences	E	Table 2
RTPrimerDB identification number	D	Table 2
Probe sequences	D	n/a
Location and identity of any modifications	E	No modifications were done
Manufacturer of oligonucleotides	D	Generi Biotech, Hradec Králové, Czech Republic
Purification method	D	desalted, microRNA RT primers OPC purified
**qPCR protocol**		
Complete reaction conditions	E	PCR reactions were performed in an CFX96 Touch Real-Time PCR Detection System (Bio-Rad) using SYBR Green I in final volume of 20 ul. Reaction mix consisted of 2 ul 10X PCR Buffer, 1.4 ul 50mM MgCl2, 0.8 ul 5mM dNTP Mix, 0.6 ul SYBR Green I, 0.1 ul 5 U/ul HotGoldStar DNA Polymerase (Eurogentec core kit), 1, 0.4 or 0.2 ul of each primer (5uM forward and reverse primers) according to optimized final concentration (250, 100, 50 nM respectively-specified in Table 2) and 5 ul of diluted cDNA (respective RNA input specified in Table 2). The PCR reactions were initiated with 10 minute incubation at 95°C, followed by 40 cycles of 95°C for 10 seconds, 60°C for 40 seconds. All reactions were performed in duplicate.
Reaction volume and amount of cDNA/DNA	E	Reaction volume: 20ul; amount of cDNA: 5ul of **1/5** dilution for mRNA and microRNA; **1/100 000** and **1/10 000** for 18S in liver and small intestine respectively; **1/50** dilution for miR-122, miR-16, small RNA U6 and sno202 in liver samples and U6 in small intestine (respective RNA input specified in Table 2).
Primer, (probe), MgCl2, and dNTP concentrations	E	200uM each dNTP, 3.5mM MgCl2, 1x reaction buffer, final primer concentration given in Table 2
Polymerase identity and concentration	E	HotGoldStar 5 U/µl (Eurogentec)
Buffer/kit identity and manufacturer	E	qPCR Core kit for SYBR® Green I No ROX (10x buffer, MgCl2 50mM, dNTP mix 5mM each, HotGoldStar, SYBR® Green I stock and DMSO tubes)-Eurogentec (RT-SN10-05NR)
Exact chemical composition of the buffer	D	-
Additives (SYBR Green I, DMSO, and so forth)	E	SYBR Green I diluted in DMSO, both components of the qPCR core kit
Manufacturer of plates/tubes and catalog number	D	8-strips, flat cap (BioRad)
Complete thermocycling parameters	E	Initial denaturation: 95°C for 10 minute, then 40 cycles at 95°C for 10 seconds, 60°C for 40 seconds
Reaction setup (manual/robotic)	D	manual
Manufacturer of qPCR instrument	E	Bio-Rad

qPCR validation		
Evidence of optimization (from gradients)	D	-
Specificity (gel, sequence, melt, or digest)	E	Melting curve analysis, ramping from 55°C to 95°C, in 0.5°C steps where fluorescence data are measured every 30s (measured melting temperature values are provided in Table 2, curves in Figure S1). Gene-specific amplification was confirmed by a single band in 2% agarose gel electrophoresis stained with SYBR Safe. No template controls (no cDNA in PCR) were run for each gene to detect unspecific amplification and primer dimerization.
For SYBR Green I, Cq of the NTC	E	The signal of the amplification plot was late (Cq>34). The difference between the negative control and all the cDNA sample was > 9 cycles. For most of the microRNA in the NTC samples was not detected any Cq or the difference between samples and negative control was >9 cycles.
Calibration curves with slope and y intercept	E	GAPDH: y = -3.35198x + 38.334; ACTB: y = -3.4641x + 39.122; 18S: y = -3.3416x + 37.47; HPRT1:y = -3.4916x + 37.218; B2M: y = -3.2104x + 36.753; RPlP0: y = -3.4148x + 37.857; HMBS: y = -3.5292x + 37.545; NQO1: y = -3.36x + 38.376
		miR-16: y=-3.2705x+35.053; miR-19a: y=-3.3738x+40.632; miR-122: y=-3.317x+31.444; miR-142: y=-3.3061x+45.299; miR-143: y=-3.2655x+38.496; miR-186: y=-3.2742x+40.468; miR-200a: y=-3.1331x+38.562; sno202: y=-3.491x+37.062; sno234: y=-3.5658x+37.548; U6: y=-3.301x+27.541; mir-29b: y=-3.3096x+39.958; miR-221: y=-3.4184x+39.321;
PCR efficiency calculated from slope	E	GAPDH: 92.4%; ACTB: 94.4%; 18S: 99.2%; HPRT1: 93.4%; B2M: 104.9%; RPlP0: 96.3%; HMBS: 92.0%; NQO1: 98.4%;
		miR-16: 102.2%; miR-19a: 97.9%; miR-122: 100.2%; miR-142: 100.7%; miR-143: 102.4%; miR-186: 102.0%; miR-200a: 108.5%; sno202: 93.4%; sno234: 90.7%; U6: 100.9%; mir-29b: 100.5%; miR-221: 96.1%;
CIs for PCR efficiency or SE	D	-
r2 of calibration curve	E	**GAPDH:** 0.9904; **ACTB:** 0.9982; **18S:** 0.9965 ;**HPRT1:** 0.9863; **B2M:** 0.9985; **RPlP0:** 0.9912; **HMBS:** 0.9742; **NQO1:** 0.9923;
		**miR-16:** 0.9991; **miR-19a:** 0.9986; **miR-122:** 0.9996; **miR-142:** 0.9822; **miR-143:** 0.993; **miR-186:** 0.9983; **miR-200a:** 0.9993; **sno202:** 0.9966; **sno234:** 0.9995; **U6:** 0.9985; **mir-29b:** 0.9991; **miR-221:** 0.9992;
Linear dynamic range	E	The linear dynamic range was considered taking into account the linearity of the standard curves; For mRNA: from 1/5 dilution of cDNA to 1/625 dilution; For microRNA: from 1/5 dilution of cDNA to 1/50000 dilution except low expressed microRNAs: miR-142, miR-186 linear range from 1/5 dilution of cDNA to 1/625 dilution.
Cq variation at LOD	E	not detected
CIs throughout range	D	-
Evidence for LOD	E	not detected, the dilutions of cDNA performed in linear range defined by standard curve
If multiplex, efficiency and LOD of each assay	E	n/a

Data analysis		
qPCR analysis program (source, version)	E	Bio-Rad CFX Manager
Method of Cq determination	E	The threshold is set manualy to the level where the fluorescence rises above detection limit. The threshold specify Cq values.
Outlier identification and disposition	E	None of the Cq values was discarded
Results for NTCs	E	The signal of the amplification plot was late (Cq>34). The difference between the negative control and all the cDNA sample was > 9 cycles. For most of the microRNA in the NTC samples was not detected any Cq or the difference between samples and negative control was >9 cycles.
Justification of number and choice of reference genes	E	This is a study for the selection of reference genes
Description of normalization method	E	Described in text
Number and concordance of biological replicates	D	Three biological replicates
Number and stage of technical replicates	E	qPCR reactions were performed in duplicate
Repeatability (intraassay variation)	E	Mean standard deviation of duplicates: 0.15
Reproducibility (interassay variation, CV)	D	-
Power analysis	D	-
Statistical methods for results significance	E	two-way ANOVA with multiple comparisons and uncorrected Fishers LSD test
Software (source, version)	E	GraphPad Prism 6
Cq or raw data submission with RDML	D	-
