# Supplementary material for: Reference Genes for Real-Time PCR Quantification of Messenger RNAs and MicroRNAs in Mouse Model of Obesity
Source: PLoS One. 2014 Jan 17;9(1):e86033. doi: 10.1371/journal.pone.0086033 (PMC3895018; doi:10.1371/journal.pone.0086033)
Supplement: Data S1 — Description of 3′:5′assay. (PDF) [file pone.0086033.s003.pdf]

Data S1

3´: 5´assay / Determination of mRNA integrity

PCR-based 3´: 5´assay is an RNA quality assessment tool. It is based on the evidence that cDNA yield from sequences near 5´end of the gene of partially degraded mRNAs is significantly lower than that from sequences near 3´end. In our 3´: 5´assay, GAPDH was used as the target sequence.

All isolated RNAs were tested as follows: cDNA was prepared similarly as described in Methods, except for using oligo(dT)$_{18}$ primer instead of random hexamers. Two independent qPCR assays were used to quantitate the levels of two target amplicons designed on different regions of GAPDH gene. One primer set was designed to amplify region near 5´end of the GAPDH gene (obtained from PrimerBank, ID:6679937a1, F: 5´-*AGGTCGGTGTGAACGGATTTG*-3´, R: 5´-*TGTAGACCATGTAGTTGAGGTCA-3´*) and the second set was designed near the 3´end of the GAPDH gene (obtained from RTPrimerDB, ID:8591, F:5´-*CTCCCACTCTTCCACCTTCG*-3´, R:5´-*CCACCACCCTGTTGCTGTAG*-3´) (Fig. 1). The ratio of amplicons relative quantity reflects the RNA integrity reversely transcribed from 3´ to 5´ into the cDNA along the entire length of the transcript. Consequently a 3´: 5´ratio of around 1 indicates high integrity, as proposed by Nolan et al., and values greater than 5 suggest RNA degradation. To perform this calculation, efficiencies of both qPCR reactions must be the same [1]. The ratio is calculated according to comparative Ct method as follows: ΔCq method (2^(-ΔCq)), ΔCq= Cq(3´)-Cq(5´) (Table 1, 2) [2].

Figure 1. **Determination of amplification efficiency using standard curves**.

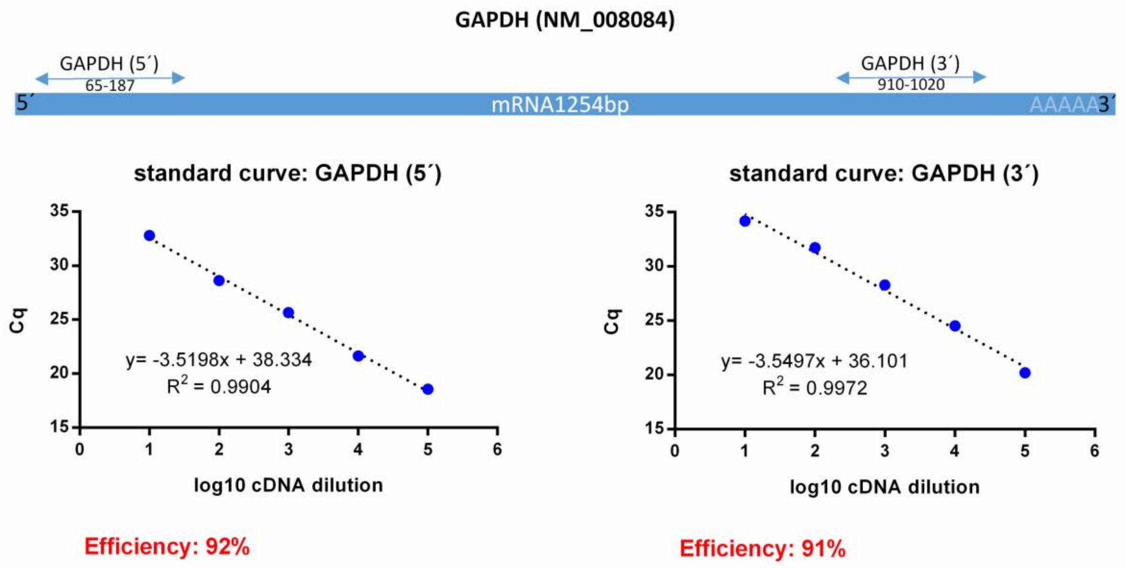

Table 1. 3´: 5´ratio calculation in the liver samples.

N	Cq (3´)*	Cq(5´)*	ΔCq[#]	3´:5´ratio
1	15,20	16,38	-1,18	2,26
2	15,36	17,16	-1,80	3,47
3	15,02	16,81	-1,79	3,45
4	15,60	17,24	-1,64	3,12
5	15,53	16,50	-0,97	1,96
6	16,11	17,34	-1,23	2,35
7	15,46	16,89	-1,43	2,70
8	15,37	17,16	-1,79	3,45
9	15,53	17,22	-1,69	3,22
10	14,66	15,26	-0,60	1,51
11	14,38	15,81	-1,44	2,71
12	14,58	16,03	-1,45	2,74

* average of duplicates of qPCR quantificatin cycles (Cq)

[#] ΔCq=Cq(3´)-Cq(5´)

» 3´:5´ratio = 2^(-ΔCq)

Table 2. 3´: 5´ratio calculation in the small intestine samples.

N	Cq (3´)*	Cq(5´)*	ΔCq[#]	3´:5´ratio»
1	16,61	18,38	-1,77	3,41
2	17,86	20,27	-2,41	5,30
3	17,20	19,33	-2,13	4,38
4	17,66	20,04	-2,39	5,22
5	17,15	18,93	-1,79	3,45
6	17,22	19,27	-2,05	4,13
7	16,69	17,56	-0,87	1,83
8	17,88	19,79	-1,91	3,76
9	17,39	19,00	-1,61	3,05
10	16,75	18,17	-1,43	2,69
11	17,71	19,59	-1,88	3,68
12	17,81	19,54	-1,73	3,32

* average of duplicates of qPCR quantificatin cycles (Cq)

[#] ΔCq=Cq(3´)-Cq(5´)

» 3´:5´ratio=2^(-ΔCq)

1. Nolan T, Hands RE, Bustin SA (2006) Quantification of mRNA using real-time RT-PCR. Nature Protocols 1: 1559-1582.
2. Schmittgen TD, Livak KJ (2008) Analyzing real-time PCR data by the comparative C-T method. Nature Protocols 3: 1101-1108.
