# Supplementary material for: Reference Genes for Real-Time PCR Quantification of Messenger RNAs and MicroRNAs in Mouse Model of Obesity
Source: PLoS One. 2014 Jan 17;9(1):e86033. doi: 10.1371/journal.pone.0086033 (PMC3895018; doi:10.1371/journal.pone.0086033)
Supplement: Data S2 — Stability values of the candidate reference genes for mRNA and miRNA normalization calculated by NormFinder with different grouping applied. (PDF) [file pone.0086033.s004.pdf]

Data S2

Candidate reference genes for mRNA normalization

Normfinder version 0.953

http://moma.dk/normfinder-software

liver: "obesity grouping" obese versus lean mice (independet of treatment)

Gene name	Stability value
18S	0,162
HPRT1	0,144
B2M	0,176
RPIP0	0,291
GAPDH	0,491
ACTB	0,424
HMBS	0,339

**Best gene**	HPRT1
**Stability value**	0,144
**Best combination of two genes**	18S and HPRT1
**Stability value for best combination of two genes**	0,087

Intragroup variation

Group identifier	1	2
18S	0,074	0,013
HPRT1	0,011	0,005
B2M	0,266	0,005
RPIP0	0,038	0,196
GAPDH	0,103	1,493
ACTB	0,714	0,031
HMBS	0,255	0,346

Intergroup variation

Group identifie	1	2
18S	0,059	-0,059
HPRT1	-0,095	0,095
B2M	-0,025	0,025
RPIP0	0,171	-0,171
GAPDH	0,329	-0,329
ACTB	-0,298	0,298
HMBS	-0,141	0,141

liver: "treatment grouping" (catechin fed mice versus mice on normal diet)

Gene name	Stability value
18S	0,124
HPRT1	0,117
B2M	0,184
RPIP0	0,262
GAPDH	0,511
ACTB	0,363
HMBS	0,314

**Best gene**	HPRT1
**Stability value**	0,117
**Best combination of two genes**	18S and HPRT1
**Stability value for best combination of two genes**	0,086

Intragroup variation

Group identifier	1	2
18S	0,112	0,006
HPRT1	0,022	0,008
B2M	0,133	0,089
RPIP0	0,210	0,018
GAPDH	0,069	1,828
ACTB	0,345	0,413
HMBS	0,115	0,558

Intergroup variation

Group identifie	1	2
18S	0,019	-0,019
HPRT1	-0,053	0,053
B2M	-0,015	0,015
RPIP0	-0,171	0,171
GAPDH	0,350	-0,350
ACTB	-0,254	0,254
HMBS	0,125	-0,125

liver: 4 separate groups

Gene name	Stability value
18S	0,203
HPRT1	0,190
B2M	0,303
RPlP0	0,303
GAPDH	0,505
ACTB	0,503
HMBS	0,432

**Best gene**	HPRT1
**Stability value**	0,190
**Best combination of two genes**	18S and HPRT1
**Stability value for best combination of two genes**	0,145

Intragroup variation

Group identifier	1	2	3	4
18S	0,119	0,130	0,035	0,005
HPRT1	0,022	0,042	0,023	0,003
B2M	0,300	0,003	0,295	0,003
RPlP0	0,075	0,096	0,009	0,064
GAPDH	0,103	0,093	0,229	2,249
ACTB	0,344	0,031	1,508	0,002
HMBS	0,127	0,086	0,659	0,747

Intergroup variation

Group identifier	1	2	3	4
18S	0,136	-0,096	-0,036	-0,005
HPRT1	-0,108	0,027	-0,078	0,160
B2M	0,129	-0,118	-0,213	0,202
RPlP0	0,125	-0,477	0,226	0,126
GAPDH	0,318	0,255	0,343	-0,916
ACTB	-0,585	0,114	0,054	0,418
HMBS	-0,015	0,295	-0,295	0,015

Candidate reference genes for mRNA normalization

Normfinder version 0.953

http://moma.dk/normfinder-software

small intestine: "obesity grouping" obese versus lean mice (independet of treatment)

Gene name	Stability value
18S	0,088
HPRT1	0,083
B2M	0,189
RPIP0	0,094
GAPDH	0,343
ACTB	0,206
HMBS	0,116

**Best gene**	HPRT1
**Stability value**	0,083
**Best combination of two genes**	RPIP0 and HMBS
**Stability value for best combination of two genes**	0,0681

Intragroup variation

Group identifier	1	2
18S	0,074	0,019
HPRT1	0,007	0,029
B2M	0,451	0,053
RPIP0	0,043	0,017
GAPDH	0,665	0,743
ACTB	0,394	0,129
HMBS	0,099	0,030

Intergroup variation

Group identifie	1	2
18S	0,006	-0,006
HPRT1	-0,114	0,114
B2M	-0,060	0,060
RPIP0	-0,159	0,159
GAPDH	-0,014	0,014
ACTB	0,171	-0,171
HMBS	0,170	-0,170

small intestine: "treatment grouping" (catechin fed mice versus mice on normal diet)

Gene name	Stability value
18S	0,127
HPRT1	0,147
B2M	0,253
RPIP0	0,161
GAPDH	0,362
ACTB	0,289
HMBS	0,168

**Best gene**	18S
**Stability value**	0,127
**Best combination of two genes**	18S and HMBS
**Stability value for best combination of two genes**	0,120

Intragroup variation

Group identifier	1	2
18S	0,004	0,083
HPRT1	0,074	0,022
B2M	0,184	0,252
RPIP0	0,038	0,097
GAPDH	0,496	0,772
ACTB	0,003	0,435
HMBS	0,103	0,126

Intergroup variation

Group identifie	1	2
18S	0,053	-0,053
HPRT1	0,061	-0,061
B2M	-0,158	0,158
RPIP0	0,061	-0,061
GAPDH	0,206	-0,206
ACTB	-0,235	0,235
HMBS	0,012	-0,012

small intestine: 4 separate groups

Gene name	Stability value
18S	0,109
HPRT1	0,092
B2M	0,258
RPlP0	0,098
GAPDH	0,468
ACTB	0,198
HMBS	0,152

**Best gene**	HPRT1
**Stability value**	0,092
**Best combination of two genes**	HPRT1 and RPlP0
**Stability value for best combination of two genes**	0,069

Intragroup variation

Group identifier	1	2	3	4
18S	0,004	0,026	0,222	0,003
HPRT1	0,069	0,003	0,024	0,026
B2M	0,414	0,020	0,583	0,059
RPlP0	0,036	0,032	0,031	0,018
GAPDH	0,068	1,137	1,170	0,698
ACTB	0,002	0,003	0,614	0,240
HMBS	0,033	0,039	0,226	0,039

Intergroup variation

Group identifier	1	2	3	4
18S	0,012	0,093	-0,001	-0,104
HPRT1	-0,132	0,254	-0,096	-0,027
B2M	-0,253	-0,062	0,132	0,183
RPlP0	-0,034	0,157	-0,284	0,161
GAPDH	0,314	0,098	-0,341	-0,071
ACTB	-0,137	-0,334	0,478	-0,007
HMBS	0,229	-0,206	0,112	-0,135
