# Supplementary material for: Reference Genes for Real-Time PCR Quantification of Messenger RNAs and MicroRNAs in Mouse Model of Obesity
Source: PLoS One. 2014 Jan 17;9(1):e86033. doi: 10.1371/journal.pone.0086033 (PMC3895018; doi:10.1371/journal.pone.0086033)
Supplement: Data S3 — BestKeeper analysis. (PDF) [file pone.0086033.s005.pdf]

Data S3

Candidate reference genes for mRNA normalization

BestKeeper version 1

http://gene-quantification.com/bestkeeper.html

liver

CP data of housekeeping Genes:

	HPRT1	B2M	18S	RPlP0	GAPDH	ACTB	HMBS
	HKG 1	HKG 2	HKG 3	HKG 4	HKG 5	HKG 6	HKG 7
n	11	11	11	11	11	11	11
geo Mean [CP]	21,46	14,15	23,68	18,44	17,86	18,88	23,34
ar Mean [CP]	21,47	14,16	23,69	18,45	17,88	18,92	23,36
min [CP]	20,68	13,19	22,77	17,17	16,74	17,15	22,04
max [CP]	22,46	14,79	25,12	19,62	20,51	21,67	25,16
std dev [± CP]	0,39	0,38	0,55	0,53	0,71	0,96	0,82
CV [% CP]	1,82	2,71	2,32	2,89	3,97	5,09	3,51
min [x-fold]	-1,67	-1,99	-1,86	-2,35	-2,17	-3,14	-2,34
max [x-fold]	1,93	1,58	2,71	2,21	6,27	6,34	3,27
std dev [± x-fold]	1,29	1,29	1,43	1,42	1,60	1,88	1,71

Pearson correlation coefficient (r)

vs.	HKG 1	HKG 2	HKG 3	HKG 4	HKG 5	HKG 6	HKG 7
HKG 2	0,761	-	-	-	-	-	-
p-value	0,007	-	-	-	-	-	-
HKG 3	0,673	0,537	-	-	-	-	-
p-value	0,023	0,088	-	-	-	-	-
HKG 4	0,512	0,610	0,636	-	-	-	-
p-value	0,107	0,046	0,036	-	-	-	-
HKG 5	-0,294	-0,476	-0,081	-0,367	-	-	-
p-value	0,382	0,140	0,816	0,268	-	-	-
HKG 6	0,857	0,461	0,688	0,528	-0,189	-	-
p-value	0,001	0,153	0,019	0,094	0,576	-	-
HKG 7	0,608	0,338	0,670	0,317	-0,428	0,774	-
p-value	0,047	0,308	0,024	0,343	0,189	0,005	-
BestKeeper vs.	HKG 1	HKG 2	HKG 3	HKG 4	HKG 5	HKG 6	HKG 7
coeff. of corr. [r]	0,870	0,617	0,878	0,660	-0,092	0,923	0,717
p-value	0,001	0,043	0,001	0,027	0,786	0,001	0,013

Regression Analysis: HKG vs. BestKeeper

	HPRT1	B2M	18S	RPlP0	GAPDH	ACTB	HMBS
	HKG 1 vs. BK	HKG 2 vs. BK	HKG 3 vs. BK	HKG 4 vs. BK	HKG 5 vs. BK	HKG 6 vs. BK	HKG 7 vs. BK
coeff. of corr. [r]	0,87	0,62	0,88	0,66	-0,09	0,92	0,72
coeff. of det. [r^2]	0,76	0,38	0,77	0,44	0,01	0,85	0,51
intercept [CP]	4,46	2,70	-0,60	0,95	21,49	-26,89	-4,44
slope [CP]	0,88	0,59	1,25	0,90	-0,19	2,36	1,43
SE [CP]	±0,27	±0,41	±0,371	±0,558	±1,098	±0,535	±0,758
p-value	0,001	0,043	0,001	0,027	0,786	0,001	0,013
Power of HKG [x-fold]	1,78	1,53	2,36	1,83	0,88	4,77	2,54

small intestine

CP data of housekeeping Genes:

	HPRT1	B2M	18S	RPlP0	GAPDH	ACTB	HMBS
	HKG 1	HKG 2	HKG 3	HKG 4	HKG 5	HKG 6	HKG 7
n	12	12	12	12	12	12	12
geo Mean [CP]	25,41	16,59	20,03	19,06	21,25	21,48	29,66
ar Mean [CP]	25,42	16,64	20,05	19,07	21,29	21,53	29,69
min [CP]	23,73	14,02	18,86	18,14	18,85	18,22	27,24
max [CP]	26,75	18,58	21,52	20,42	23,89	23,60	31,44
std dev [± CP]	0,82	1,00	0,72	0,59	1,10	1,14	0,97
CV [% CP]	3,22	6,01	3,60	3,10	5,15	5,29	3,25
min [x-fold]	-3,01	-6,37	-2,24	-1,86	-5,29	-8,68	-4,85
max [x-fold]	2,41	4,16	2,79	2,50	6,24	4,09	3,18
std dev [± x-fold]	1,71	1,93	1,61	1,47	2,06	2,12	1,89

Pearson correlation coefficient (r)

vs.	HKG 1	HKG 2	HKG 3	HKG 4	HKG 5	HKG 6	HKG 7
HKG 2	0,759	-	-	-	-	-	-
p-value	0,004	-	-	-	-	-	-
HKG 3	0,896	0,845	-	-	-	-	-
p-value	0,001	0,001	-	-	-	-	-
HKG 4	0,911	0,872	0,921	-	-	-	-
p-value	0,001	0,001	0,001	-	-	-	-
HKG 5	0,682	0,390	0,553	0,642	-	-	-
p-value	0,015	0,210	0,062	0,024	-	-	-
HKG 6	0,849	0,903	0,896	0,876	0,419	-	-
p-value	0,001	0,001	0,001	0,001	0,175	-	-
HKG 7	0,891	0,741	0,873	0,865	0,670	0,859	-
p-value	0,001	0,006	0,001	0,001	0,017	0,001	-
BestKeeper vs.	HKG 1	HKG 2	HKG 3	HKG 4	HKG 5	HKG 6	HKG 7
coeff. of corr. [r]	0,940	0,894	0,946	0,964	0,682	0,931	0,928
p-value	0,001	0,001	0,001	0,001	0,015	0,001	0,001

Regression Analysis: HKG vs. BestKeeper

	HPRT1	B2M	18S	RPlP0	GAPDH	ACTB	HMBS
	HKG 1 vs. BK	HKG 2 vs. BK	HKG 3 vs. BK	HKG 4 vs. BK	HKG 5 vs. BK	HKG 6 vs. BK	HKG 7 vs. BK
coeff. of corr. [r]	0,94	0,89	0,95	0,96	0,68	0,93	0,93
coeff. of det. [r^2]	0,88	0,80	0,90	0,93	0,47	0,87	0,86
intercept [CP]	6,44	-7,54	2,19	4,70	1,25	-7,86	5,33
slope [CP]	0,88	1,12	0,83	0,67	0,93	1,36	1,13
SE [CP]	±0,344	±0,607	±0,306	±0,199	±1,076	±0,576	±0,49
p-value	0,001	0,001	0,001	0,001	0,015	0,001	0,001
Power of HKG [x-fold]	1,78	2,23	1,77	1,56	1,90	2,46	2,09

Candidate reference genes for miRNA normalization

BestKeeper version 1

http://gene-quantification.com/bestkeeper.html

liver

CP data of housekeeping Genes:

	mir186	mir142	U6	sno202	miR16	mir122	sno234	mir143	mir19a	mir200a
	HKG 1	HKG 2	HKG 3	HKG 4	HKG 5	HKG 6	HKG 7	HKG 8	HKG 9	HKG 10
n	12	12	12	12	12	12	12	12	12	12
geo Mean [CP]	26,03	31,28	16,37	23,35	23,03	18,56	16,64	21,80	25,23	25,64
ar Mean [CP]	26,03	31,28	16,38	23,35	23,03	18,56	16,64	21,80	25,23	25,65
min [CP]	25,64	30,40	15,15	22,68	22,56	17,97	16,13	21,23	24,66	24,21
max [CP]	26,49	32,55	17,14	24,25	23,40	19,03	17,20	22,46	25,86	26,62
std dev [± CP]	0,28	0,54	0,41	0,49	0,18	0,34	0,24	0,34	0,31	0,62
CV [% CP]	1,06	1,73	2,50	2,10	0,80	1,85	1,46	1,56	1,22	2,41
min [x-fold]	-1,31	-1,83	-2,34	-1,59	-1,39	-1,50	-1,42	-1,49	-1,48	-2,69
max [x-fold]	1,38	2,42	1,70	1,87	1,29	1,39	1,48	1,57	1,55	1,97
std dev [± x-fold]	1,21	1,46	1,33	1,41	1,14	1,27	1,18	1,27	1,24	1,53

Pearson correlation coefficient (r)

vs.	HKG 1	HKG 2	HKG 3	HKG 4	HKG 5	HKG 6	HKG 7	HKG 8	HKG 9	HKG 10
HKG 2	0,338	-	-	-	-	-	-	-	-	-
p-value	0,281	-	-	-	-	-	-	-	-	-
HKG 3	0,154	0,717	-	-	-	-	-	-	-	-
p-value	0,635	0,009	-	-	-	-	-	-	-	-
HKG 4	0,020	-0,335	-0,534	-	-	-	-	-	-	-
p-value	0,953	0,289	0,073	-	-	-	-	-	-	-
HKG 5	-0,071	0,025	-0,307	0,676	-	-	-	-	-	-
p-value	0,823	0,938	0,332	0,016	-	-	-	-	-	-
HKG 6	0,042	0,653	0,619	-0,349	-0,200	-	-	-	-	-
p-value	0,899	0,021	0,032	0,265	0,530	-	-	-	-	-
HKG 7	0,183	-0,367	-0,204	0,736	0,381	-0,190	-	-	-	-
p-value	0,568	0,240	0,524	0,006	0,223	0,555	-	-	-	-
HKG 8	0,394	0,707	0,399	-0,227	0,095	0,383	-0,372	-	-	-
p-value	0,204	0,010	0,198	0,476	0,770	0,219	0,233	-	-	-
HKG 9	-0,308	0,136	0,132	0,429	0,404	0,160	0,066	0,292	-	-
p-value	0,332	0,676	0,683	0,165	0,192	0,621	0,838	0,355	-	-
HKG 10	0,025	0,584	0,552	-0,681	-0,356	0,777	-0,352	0,260	-0,381	-
p-value	0,938	0,047	0,063	0,015	0,258	0,003	0,262	0,415	0,223	-
BestKeeper vs.	HKG 1	HKG 2	HKG 3	HKG 4	HKG 5	HKG 6	HKG 7	HKG 8	HKG 9	HKG 10
coeff. of corr. [r]	0,331	0,844	0,731	-0,067	0,158	0,781	0,071	0,629	0,326	0,525
p-value	0,293	0,001	0,007	0,838	0,621	0,003	0,823	0,028	0,301	0,080

Regression Analysis: HKG vs. BestKeeper

	mir186	mir142	U6	sno202	miR16	mir122	sno234	mir143	mir19a	mir200a
	HKG 1 vs. BK	HKG 2 vs. BK	HKG 3 vs. BK	HKG 4 vs. BK	HKG 5 vs. BK	HKG 6 vs. BK	HKG 7 vs. BK	HKG 8 vs. BK	HKG 9 vs. BK	HKG 10 vs. BK
coeff. of corr. [r]	0,33	0,84	0,73	-0,07	0,16	0,78	0,07	0,63	0,33	0,53
coeff. of det. [r^2]	0,11	0,71	0,53	0,00	0,03	0,61	0,01	0,40	0,11	0,28
intercept [CP]	15,27	-27,57	-25,34	27,15	19,27	-12,24	14,36	-4,31	12,32	-15,81
slope [CP]	0,48	2,63	1,87	-0,17	0,17	1,38	0,10	1,17	0,58	1,85
SE [CP]	±0,315	±0,385	±0,401	±0,583	±0,241	±0,254	±0,331	±0,332	±0,385	±0,693
p-value	0,293	0,001	0,007	0,838	0,621	0,003	0,823	0,028	0,301	0,080
Power of HKG [x-fold]	1,40	6,20	3,65	0,89	1,12	2,60	1,07	2,25	1,49	3,62

small intestine

CP data of housekeeping Genes:

	mir186	mir142	U6	sno202	miR16	mir122	sno234	mir143	mir19a	mir200a
	HKG 1	HKG 2	HKG 3	HKG 4	HKG 5	HKG 6	HKG 7	HKG 8	HKG 9	HKG 10
n	12	12	12	12	12	12	12	12	12	12
geo Mean [CP]	24,15	29,48	15,97	17,88	19,77	28,82	16,71	17,31	23,41	19,31
ar Mean [CP]	24,16	29,50	15,98	17,88	19,79	28,90	16,72	17,34	23,41	19,32
min [CP]	22,73	27,85	15,19	17,47	18,60	24,16	16,10	15,03	22,51	18,33
max [CP]	25,09	31,52	17,15	18,58	21,46	32,95	17,65	18,38	24,75	20,27
std dev [± CP]	0,44	0,84	0,44	0,34	0,69	1,55	0,32	0,65	0,51	0,44
CV [% CP]	1,82	2,85	2,72	1,91	3,48	5,35	1,88	3,75	2,17	2,28
min [x-fold]	-2,67	-3,11	-1,72	-1,33	-2,25	-25,26	-1,53	-4,87	-1,86	-1,97
max [x-fold]	1,92	4,10	2,27	1,62	3,21	17,46	1,92	2,09	2,54	1,94
std dev [± x-fold]	1,36	1,79	1,35	1,27	1,61	2,92	1,24	1,57	1,42	1,36

Pearson correlation coefficient (r)

vs.	HKG 1	HKG 2	HKG 3	HKG 4	HKG 5	HKG 6	HKG 7	HKG 8	HKG 9	HKG 10
HKG 2	0,726	-	-	-	-	-	-	-	-	-
p-value	0,007	-	-	-	-	-	-	-	-	-
HKG 3	-0,396	-0,124	-	-	-	-	-	-	-	-
p-value	0,204	0,698	-	-	-	-	-	-	-	-
HKG 4	-0,173	0,073	0,767	-	-	-	-	-	-	-
p-value	0,588	0,823	0,004	-	-	-	-	-	-	-
HKG 5	0,165	0,307	0,646	0,664	-	-	-	-	-	-
p-value	0,608	0,332	0,023	0,018	-	-	-	-	-	-
HKG 6	-0,213	-0,098	0,216	0,002	-0,373	-	-	-	-	-
p-value	0,506	0,763	0,500	0,992	0,233	-	-	-	-	-
HKG 7	0,822	0,852	-0,307	-0,159	0,245	-0,343	-	-	-	-
p-value	0,001	0,001	0,332	0,621	0,442	0,277	-	-	-	-
HKG 8	0,911	0,607	-0,497	-0,329	-0,050	0,013	0,674	-	-	-
p-value	0,001	0,037	0,100	0,297	0,876	0,969	0,016	-	-	-
HKG 9	0,764	0,690	-0,396	-0,272	0,063	-0,286	0,785	0,608	-	-
p-value	0,004	0,013	0,204	0,394	0,845	0,369	0,002	0,036	-	-
HKG 10	0,848	0,638	-0,340	-0,225	0,114	-0,071	0,692	0,826	0,759	-
p-value	0,001	0,026	0,281	0,482	0,726	0,823	0,013	0,001	0,004	-
BestKeeper vs.	HKG 1	HKG 2	HKG 3	HKG 4	HKG 5	HKG 6	HKG 7	HKG 8	HKG 9	HKG 10
coeff. of corr. [r]	0,761	0,802	0,139	0,220	0,401	0,227	0,669	0,711	0,569	0,750
p-value	0,004	0,002	0,669	0,494	0,198	0,476	0,017	0,009	0,053	0,005

Regression Analysis: HKG vs. BestKeeper

	mir186	mir142	U6	sno202	miR16	mir122	sno234	mir143	mir19a	mir200a
	HKG 1 vs. BK	HKG 2 vs. BK	HKG 3 vs. BK	HKG 4 vs. BK	HKG 5 vs. BK	HKG 6 vs. BK	HKG 7 vs. BK	HKG 8 vs. BK	HKG 9 vs. BK	HKG 10 vs. BK
coeff. of corr. [r]	0,76	0,80	0,14	0,22	0,40	0,23	0,67	0,71	0,57	0,75
coeff. of det. [r^2]	0,58	0,64	0,02	0,05	0,16	0,05	0,45	0,51	0,32	0,56
intercept [CP]	-1,16	-17,29	11,73	13,07	0,85	2,22	1,34	-17,21	3,39	-4,69
slope [CP]	1,22	2,25	0,20	0,23	0,91	1,28	0,74	1,66	0,96	1,15
SE [CP]	±0,421	±0,68	±0,59	±0,416	±0,845	±2,231	±0,333	±0,666	±0,564	±0,413
p-value	0,004	0,002	0,669	0,494	0,198	0,476	0,017	0,009	0,053	0,005
Power of HKG [x-fold]	2,32	4,75	1,15	1,17	1,88	2,43	1,67	3,16	1,95	2,23
